# Supplementary material for: Wild tomato genome assemblies reveal structural variants and repeat content act as recombination barriers
Source: Nat Commun. 2026 Jun 28;17:5590. doi: 10.1038/s41467-026-74784-5 (PMC13310191; doi:10.1038/s41467-026-74784-5)
Supplement: Supplementary file 1 — Supplementary Information [file 41467_2026_74784_MOESM1_ESM.pdf]

Supplementary Fig. 1

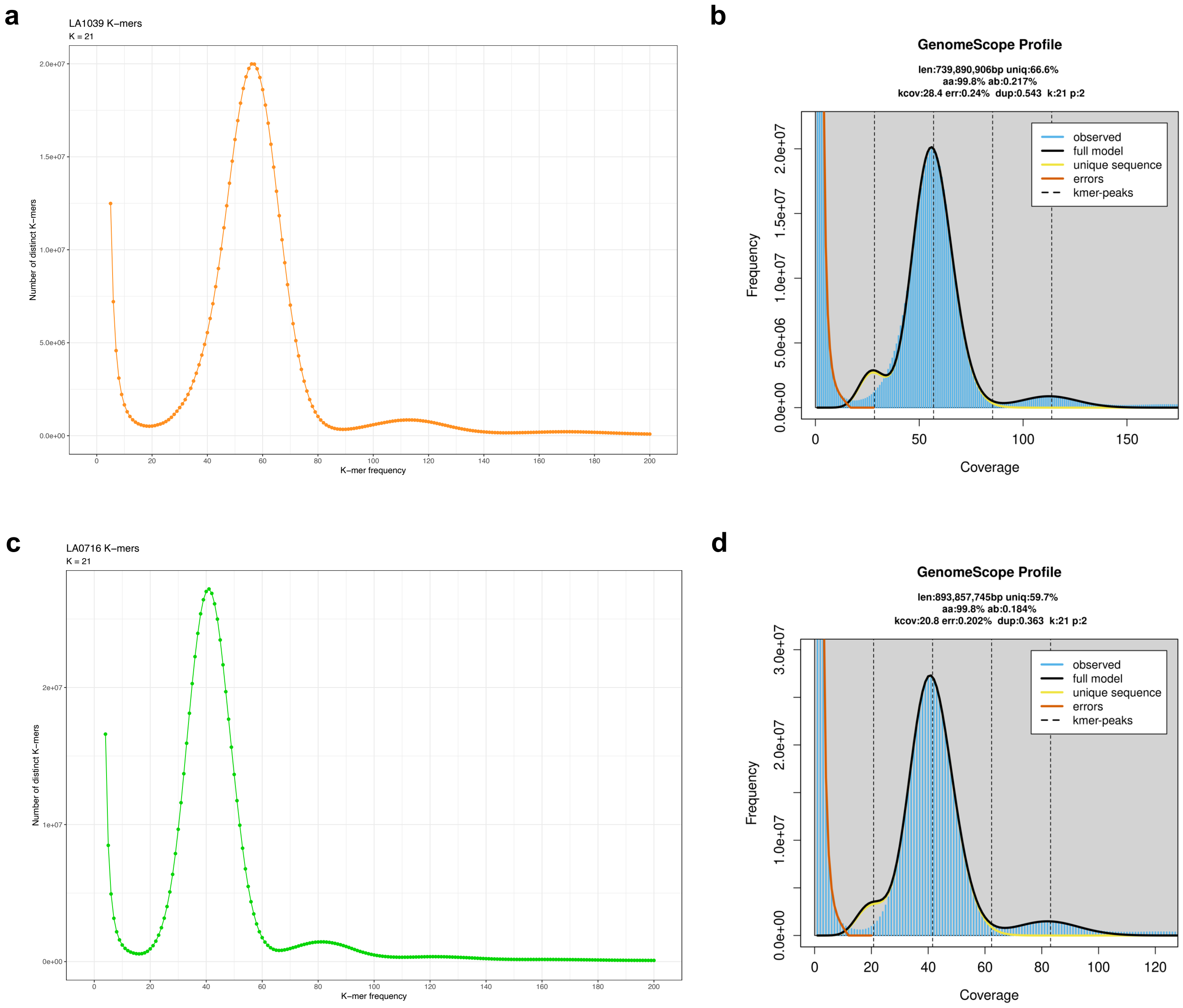

K-mer Analysis Toolkit (KAT) and GenomeScope2 k-mer spectra plots for *S. cheesemaniae* LA1039 (S.che) (**a** and **b**), and *S. pennellii* LA0716 (S.pen)(**c** and **d**).

Supplementary Fig. 2

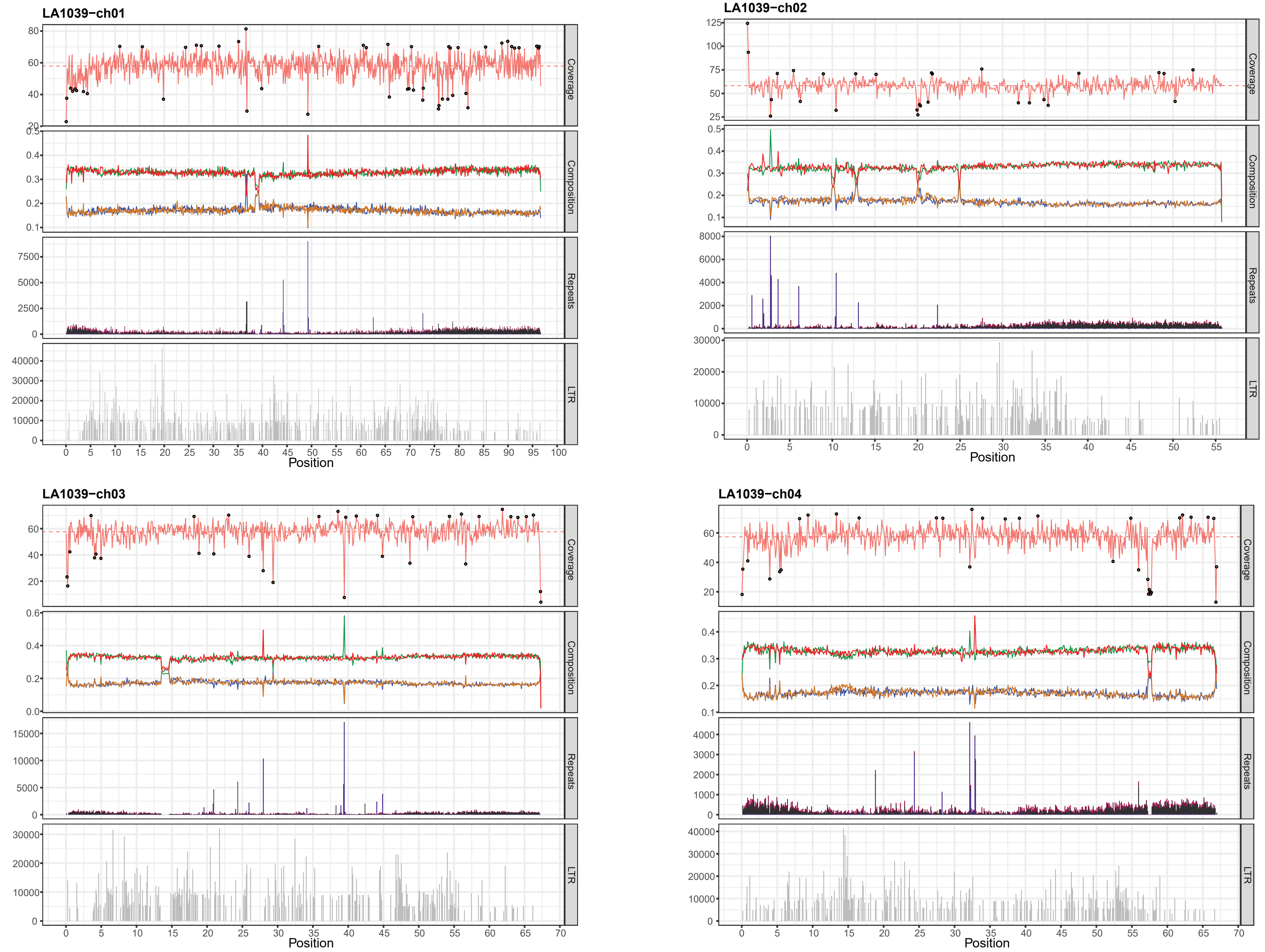

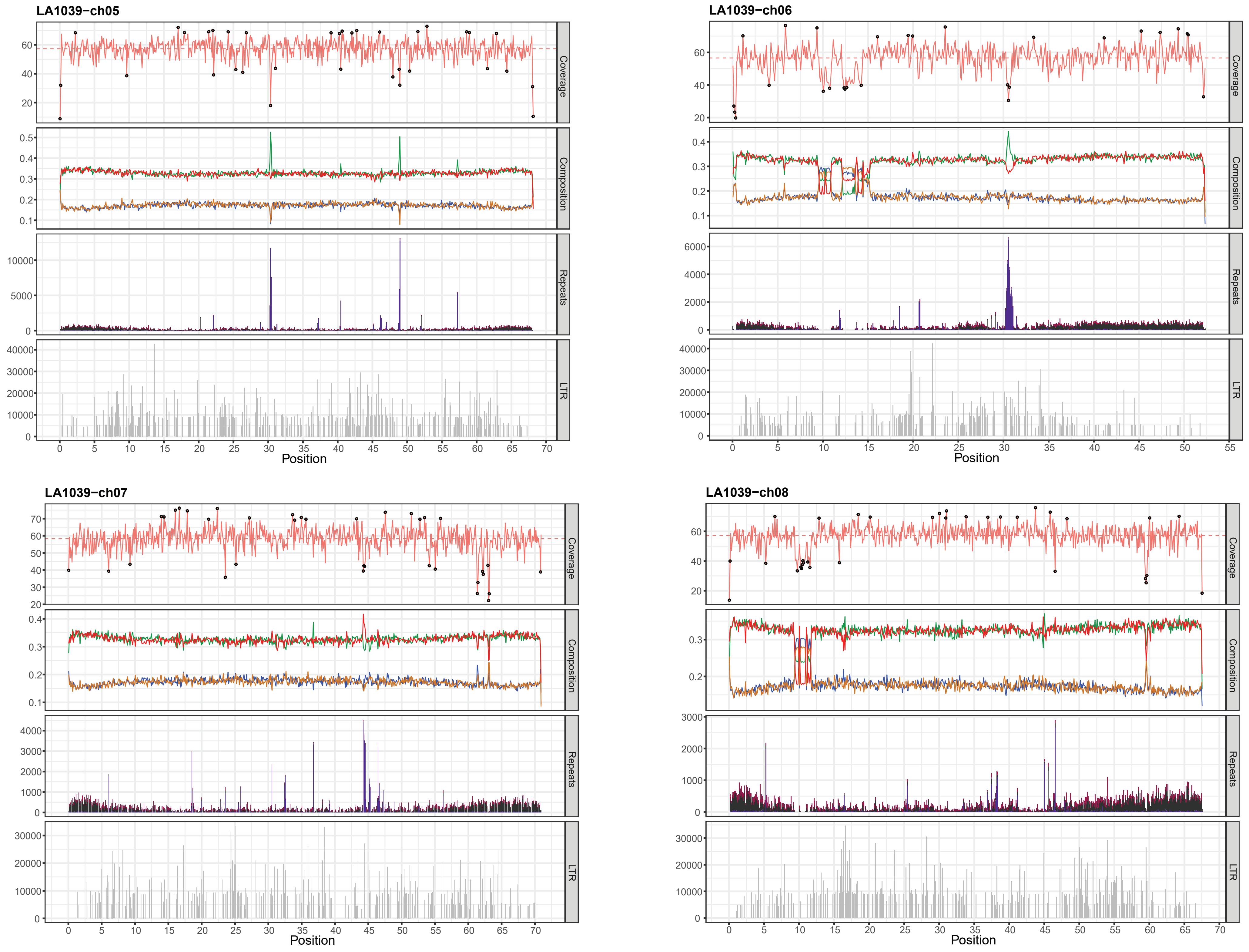

LA1039−ch05
Coverage
Composition
Repeats
LTR
Position
LA1039−ch06
Coverage
Composition
Repeats
LTR
Position
LA1039−ch07
Coverage
Composition
Repeats
LTR
Position
LA1039−ch08
Coverage
Composition
Repeats
LTR
Position

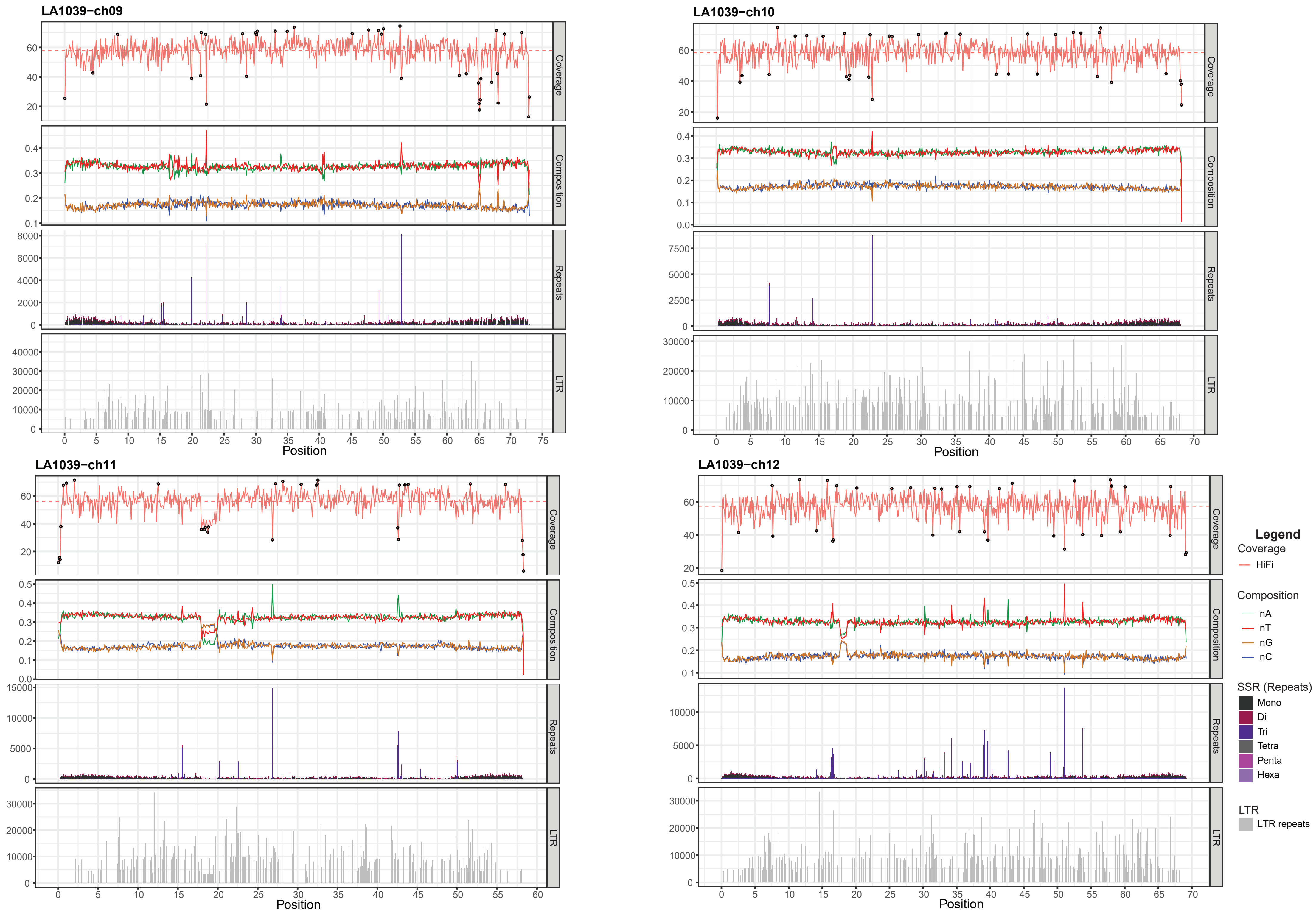

Characterization and validation of the 12 *S. cheesmaniae* LA1039 chromosomes include primary read coverage analysis (Coverage panel), nucleotide composition ratios (Composition panel), SSR Repeat analysis (Repeats panel) and LTR repeat analysis (LTR panel) in 100kb windows plotted over the genomic position on the genome. Coverage panel: dashed horizontal line represents mean coverage. Black circles represent coverage outliers (<2.5% and >97.5% percentiles). X-axis is chromosome position in megabasepair.

Supplementary Fig. 3

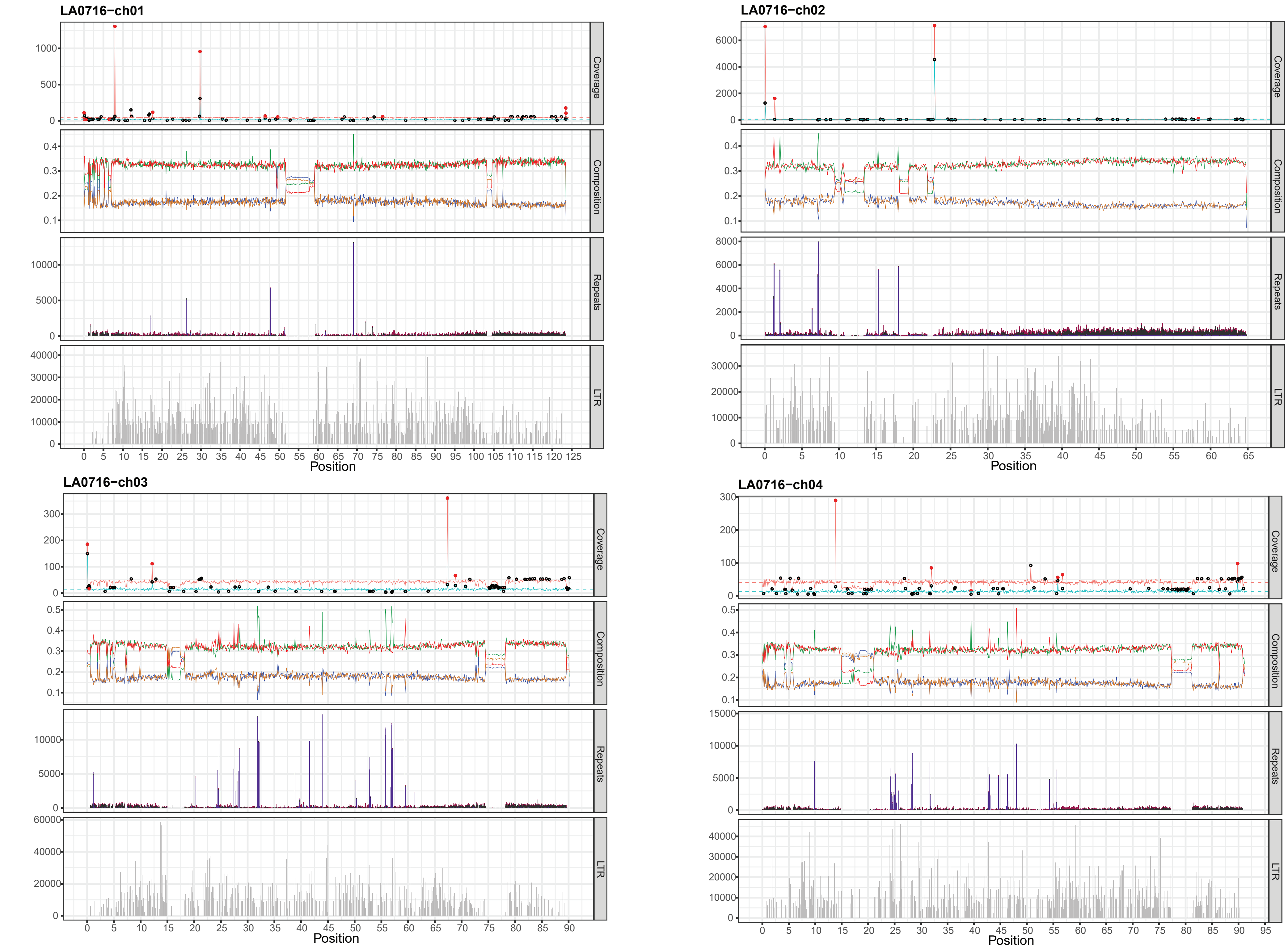

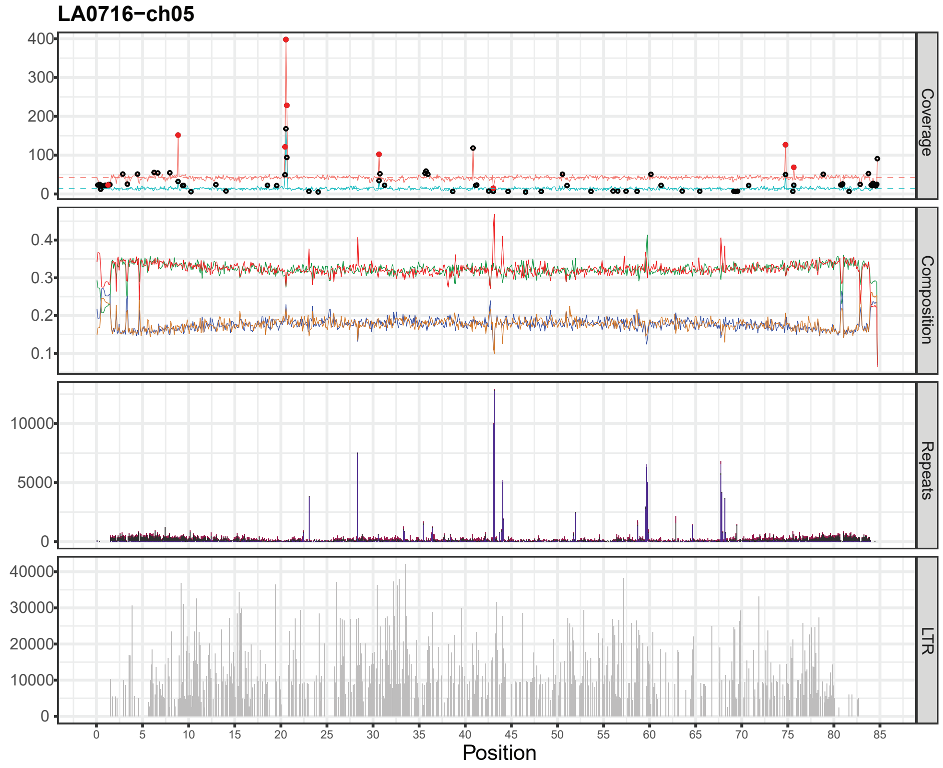
LA0716−ch05
Coverage
Composition
Repeats
LTR
Position

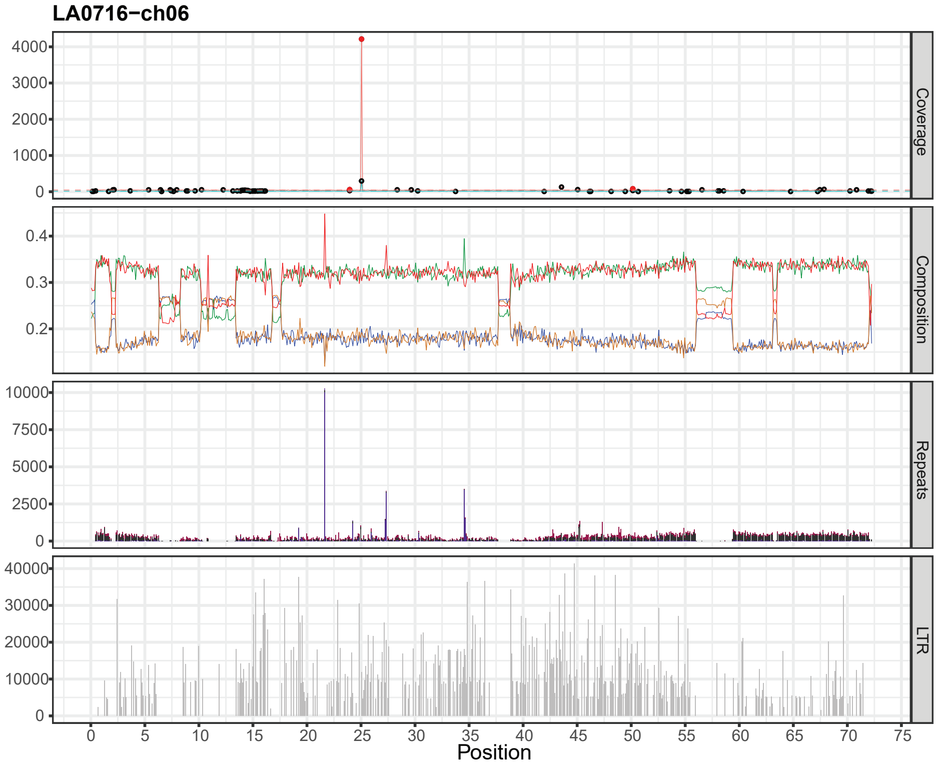
LA0716−ch06
Coverage
Composition
Repeats
LTR
Position

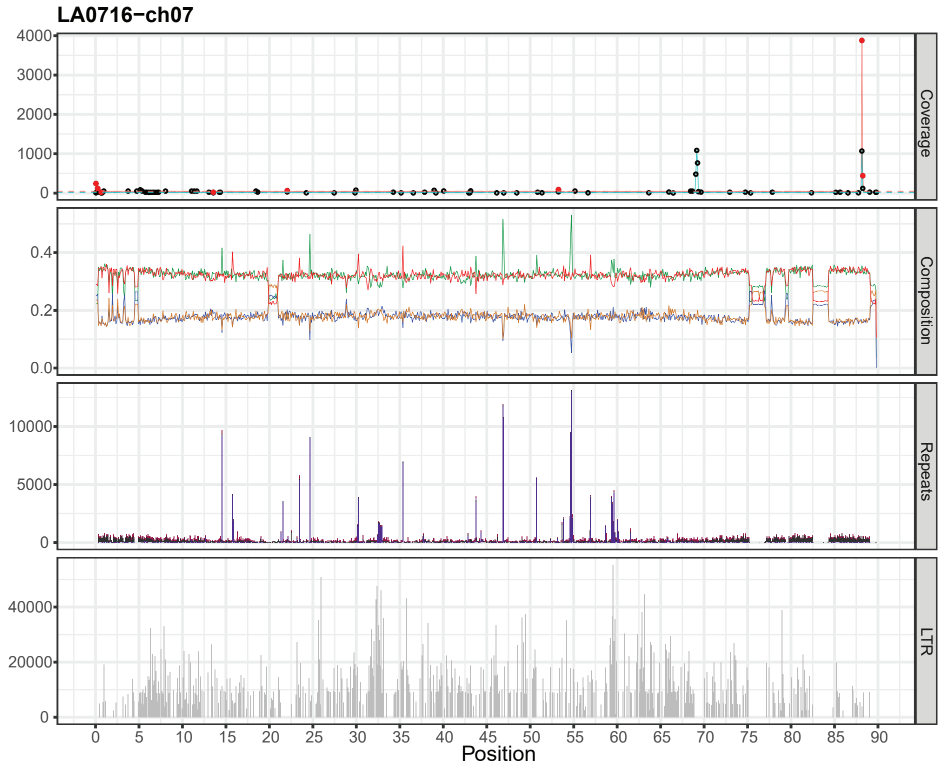
LA0716−ch07
Coverage
Composition
Repeats
LTR
Position

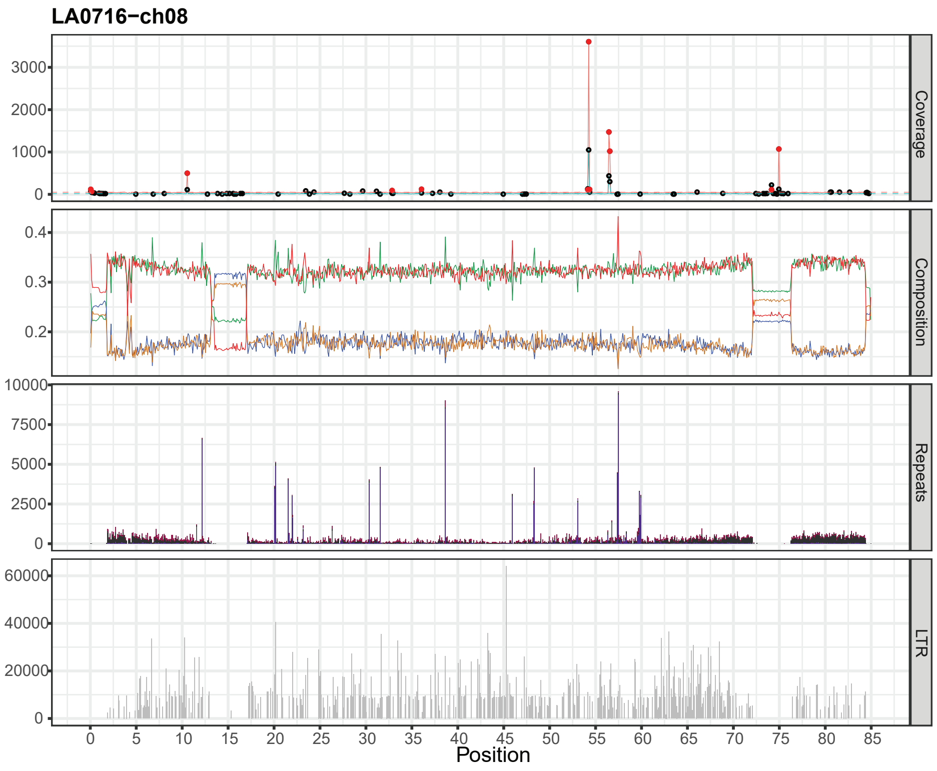
LA0716−ch08
Coverage
Composition
Repeats
LTR
Position

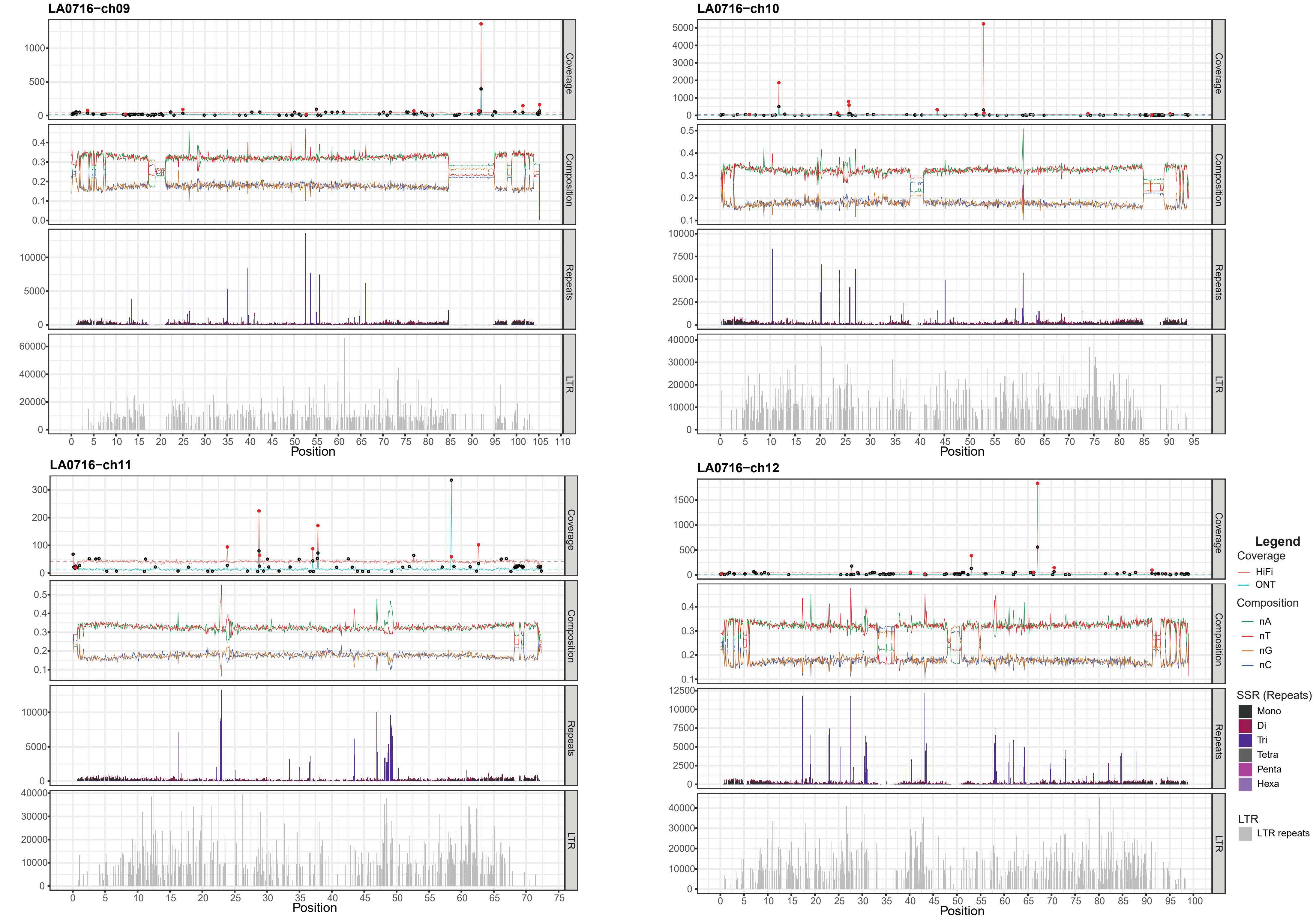

Characterization and validation of the 12 *S. pennellii* LA0716 chromosomes including primary read coverage analysis (Coverage panel), nucleotide composition ratios (Composition panel), SSR Repeat analysis (Repeats panel) and LTR repeat analysis (LTR panel) in 100kb windows plotted over the genomic position on the genome. Coverage panel: dashed horizontal line represents mean coverage. Black circles represent coverage outliers (<2.5% and >97.5% percentiles), red circles represent shared coverage outliers. X-axis is chromosome position in megabasepair.

Supplementary Fig. 4

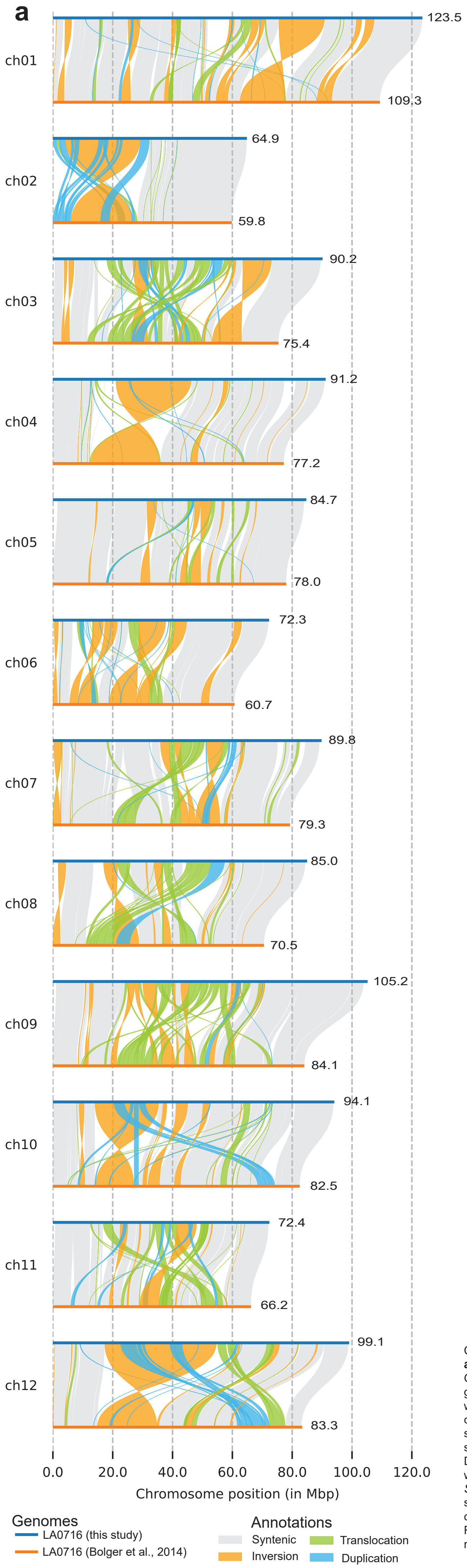

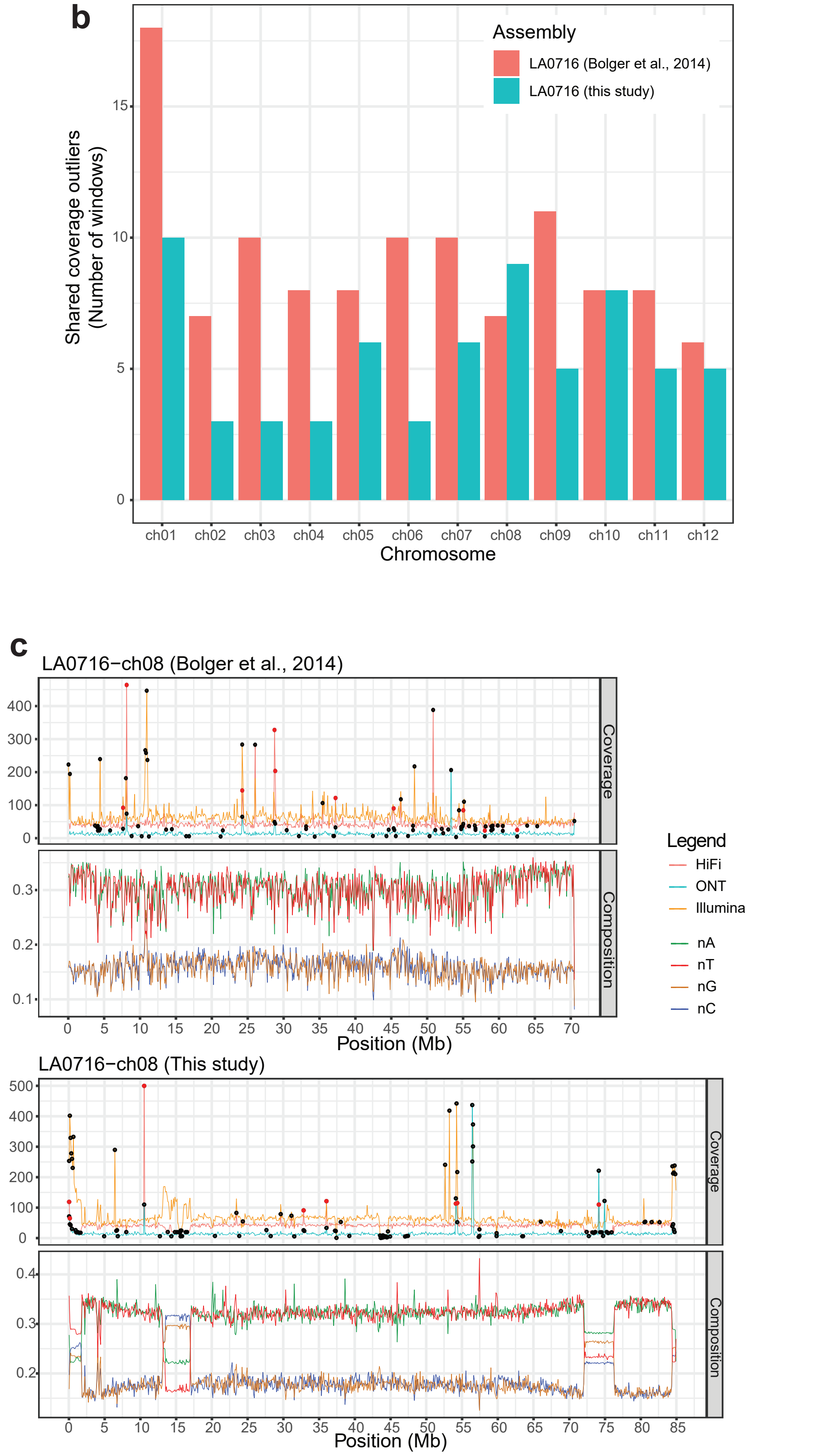

Comparison of original and new *S. pennelii* LA0716 genome assemblies
a Synteny and Rearangement plot (SyRI) between LA0716 (this study) and the 2014 LA0716 genome (Bolger et al., 2014). Genome tracks are LA0716 (this study) in blue and the 2014 LA0716 genome in orange. Annotations include syntenic regions in grey, inversions in yellow, translocations in green and duplications in blue. Non-aligned or non-syntenic regions are visualized by white gaps and can include insertions and deletions. Increased chromosome length is observed for all LA0716 (this study) chromosomes. Large genomic variation between the genomes is observed especially in pericentromeric regions and also at the start of chromosomes 6, 7 and 8. **b** Pairwise analysis of both *S. pennellii* assemblies for deviating read coverage in 100kb windows showing the number of shared windows between HiFi (this study), ONT (this study) and Illumina (Bolger et al., 2014) data. Deviating coverage is concidered coverage (<2.5% percentile and >97.5% percentile). Equal or increased numbers of windows with shared deviating coverage are observed 2014 LA0716 genome for all chromosomes except ch08. **c** Pairwise analysis of both *S. pennellii* assemblies for sequencing coverage and nucleotide composition. Sequencing coverage of HiFi (this study), ONT (this study) and Illumina (Bolger et al., 2014) data in 100kb windows. Read coverage is supportive of both assemblies. Characteristic changes in nucleotide composition patterns are lacking within the 2014 LA0716 genome, although supported by Illumina coverage. Read coverage was maximized at 500. Black circles represent coverage outliers (whereby coverage <2.5% or >97.5% percentile), red circles represent shared coverage outliers between 2 or more datasets annotated on the HiFi track.

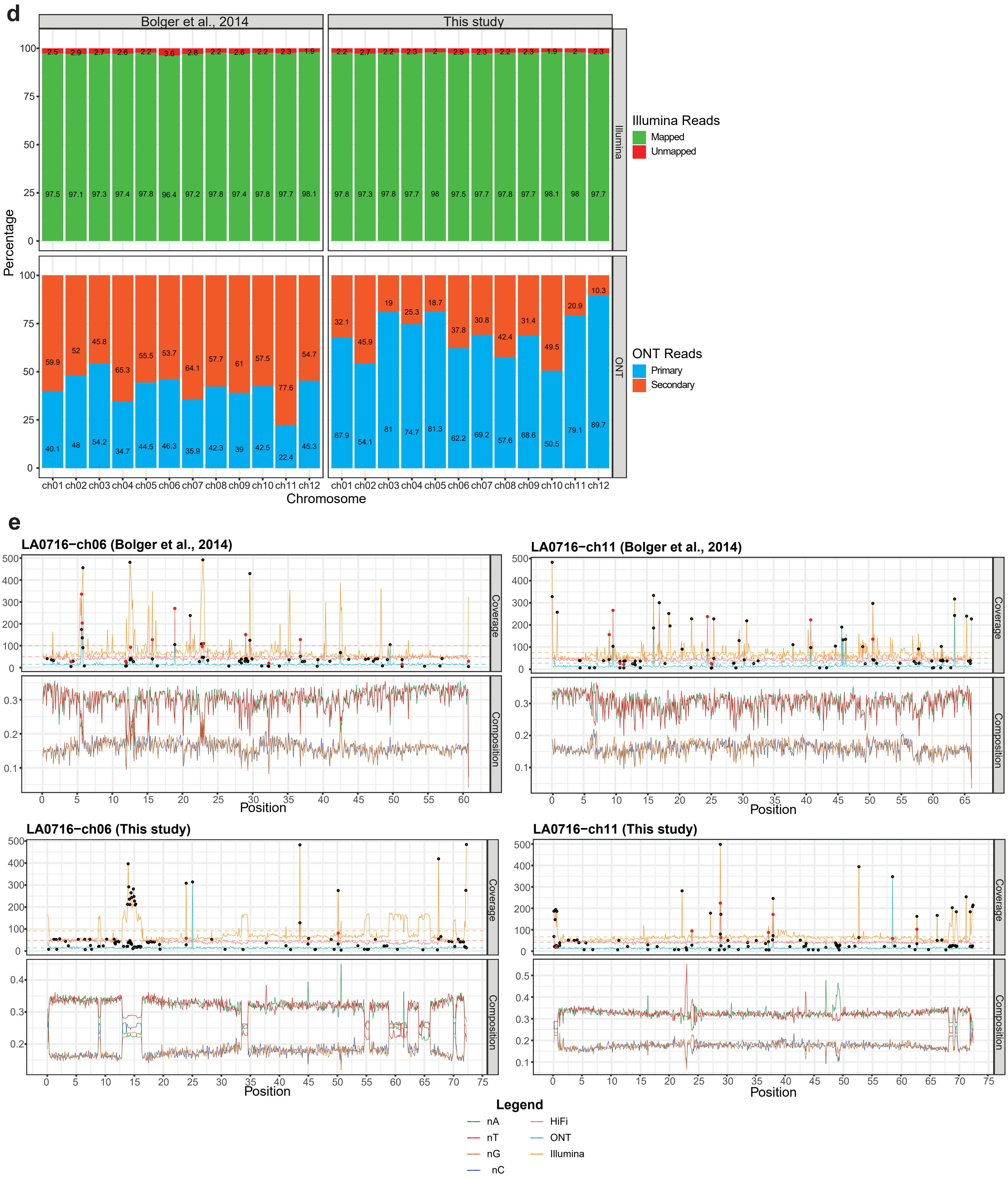

d Pairwise sequencing data (Illumina and ONT) analysis. For Illumina data (Bolger et al., 2014), percentage of mapped (green) and unmapped (red) reads to respective LA0716 genomes (this study or Bolger et al., 2014) is shown. For ONT data (This study), percentage of primary (blue) and secondary (orange) aligned reads to respecitve LA0716 genomes is shown. Increased numbers of unmapped and secondary read alignments are shown for the 2014 LA0716 genome (Bolger et al., 2014). **e** Pairwise sequencing coverage comparison of HiFi (this study), ONT (this study) and Illumina (Bolger et al., 2014) data aligned agains the two LA0716 genomes. Read coverage is supportive of both assemblies. Characteristic changes in nucleotide composition patterns are lacking within the 2014 LA0716 genome, although supported by Illumina coverage in our LA0716 genome. Black circles represent coverage outliers (whereby coverage <2.5% or >97.5% percentile), red circles represent shared coverage outliers between 2 or more datasets annotated on the HiFi track.

Supplementary Fig. 5

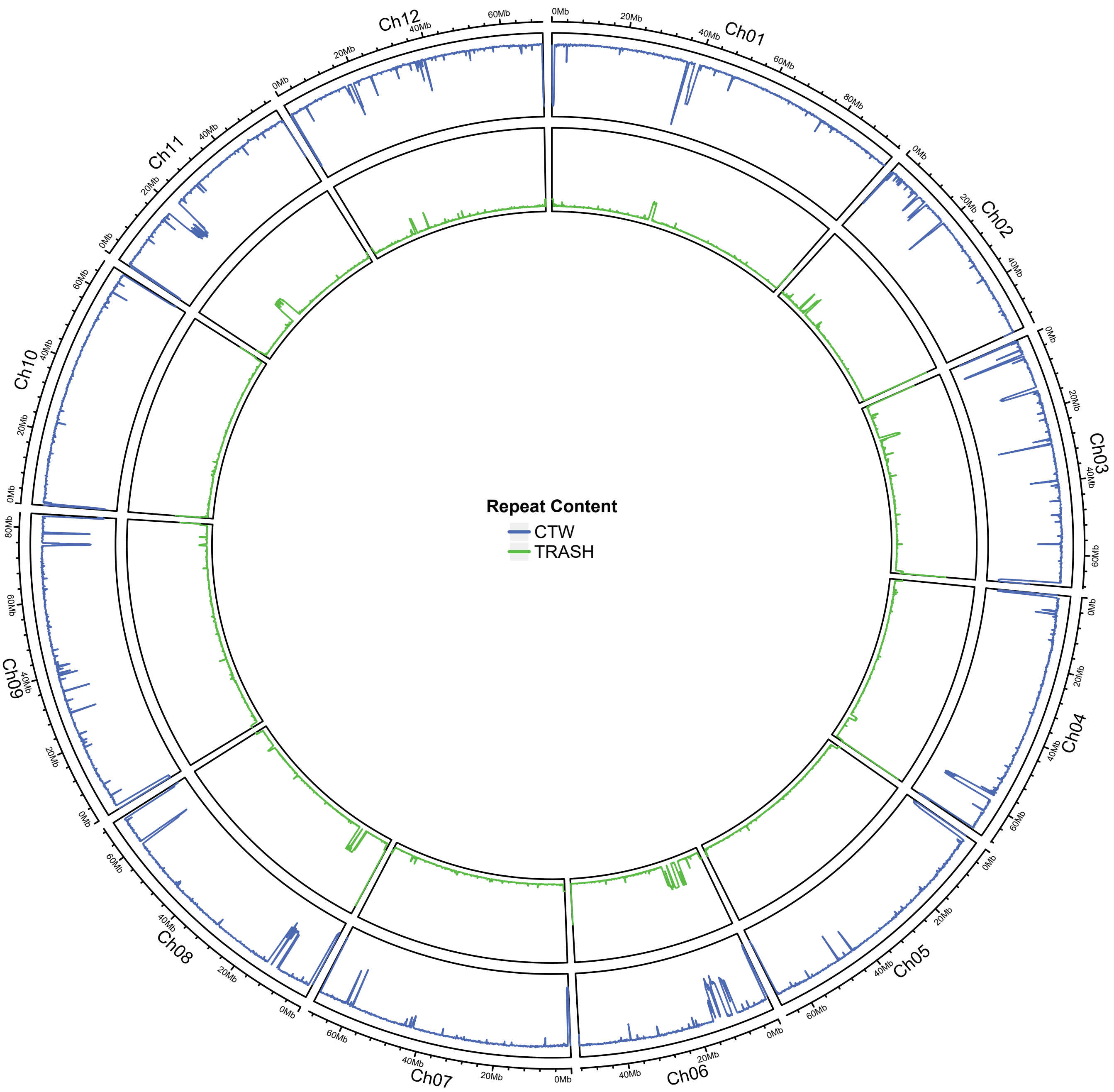

MbTMV repeat content. From outer to inner layer (in 100 Kbp windows): The log-probability of the CTW algorithm (Kontoyiannis et al., 2022) (blue line) and the abundance of monomeric repeats using TRASH (Wlodzimierz et al., 2023) (green line) for S.lycopersicum

Supplementary Fig. 6

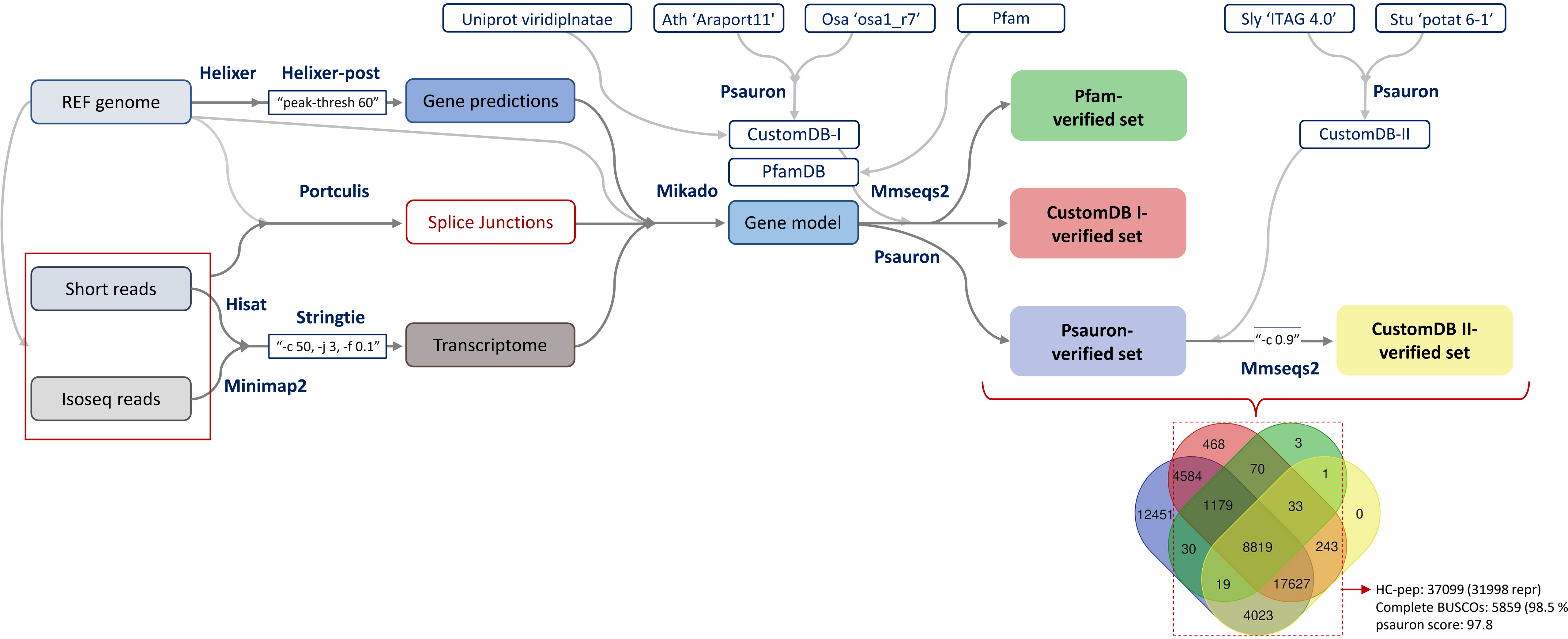

Pipeline used to develop the high-confidence gene model for LA0716. Structural gene annotation for LA0716 was conducted using an integrated pipeline combining ab-initio predictions (using Helixer v0.3.3, land_plant_v0.3_a_0080.h5) and evidence-based approaches using both short- and long-read RNA datasets (validated for splice junctions using Portcullis v1.2.4, aligned respectively using Hisat2 v2.2.1 and minimap2 v2.28, and assembled to the transcriptome using StringTie v2.2.3) via Mikado v2.3.3 to generate an initial consensus gene model, followed by a multi-layered refinement to minimize the potential false positives. Tool-specific settings are indicated in blue-bordered boxes within the schematic. Validation reference databases used for refinement are indicated in rounded blue-bordered boxes.

Supplementary Fig. 7

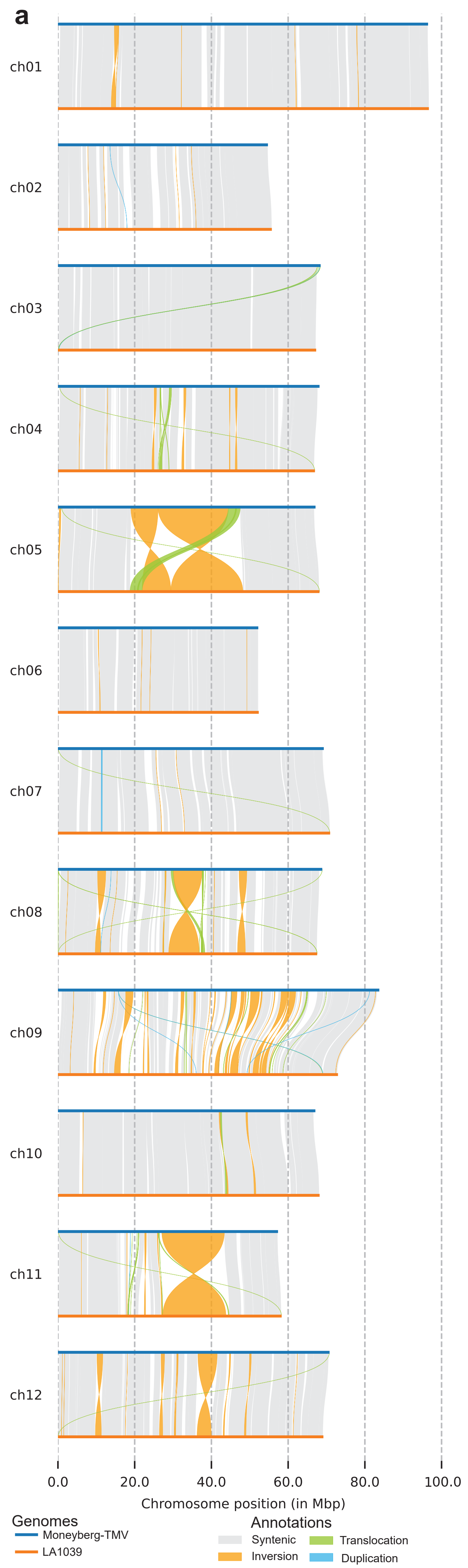

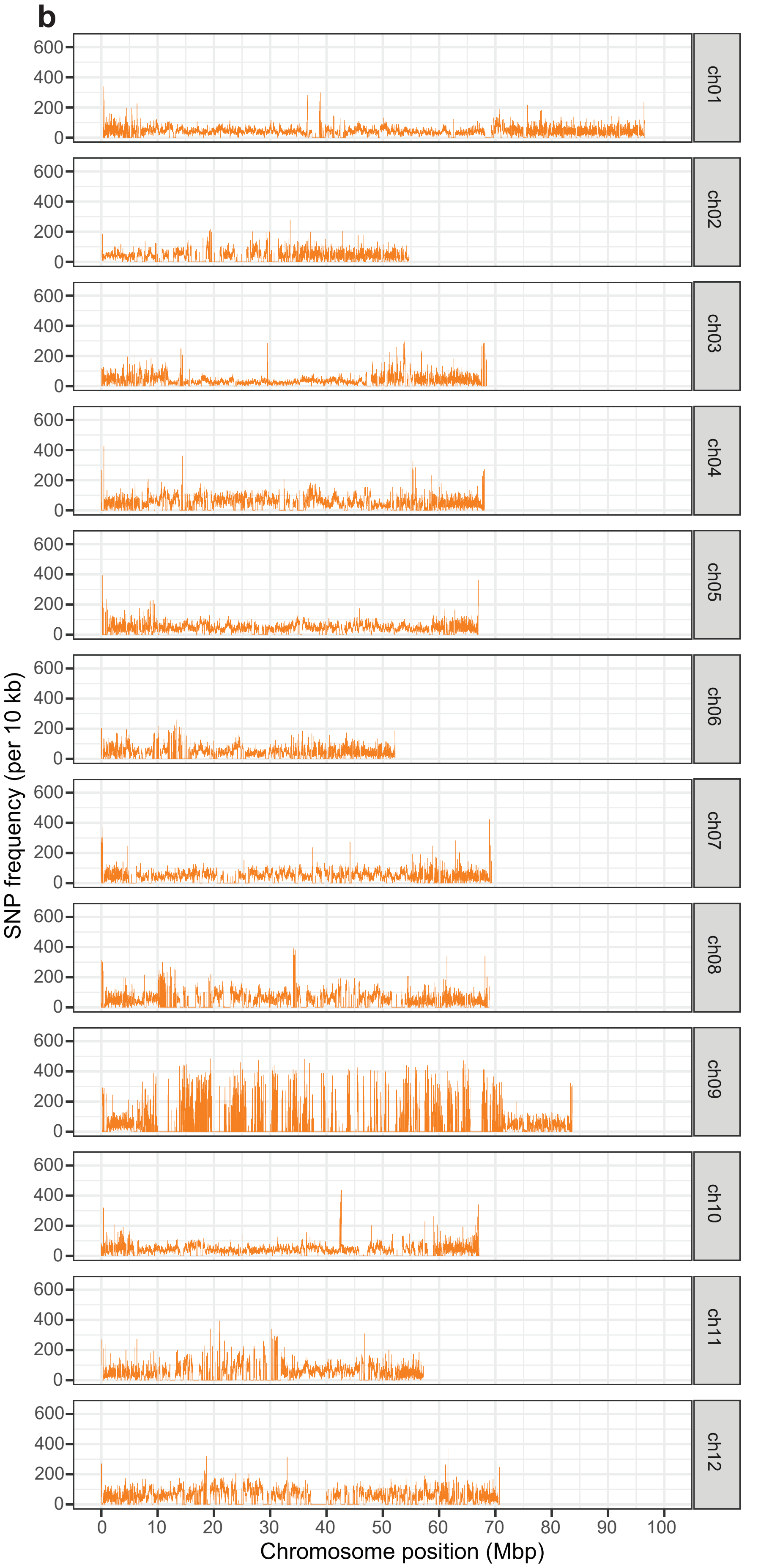

Genomic variation between Moneyberg-TMV (MbTMV) and LA1039 as identified by Synteny and Rearrangement identifier (SyRI)
a Synteny and Rearrangement plot (SyRI) between *S. lycopersicum* cv. Moneyberg-TMV and *S. cheesmaniae* LA1039. on-aligned or non-syntenic regions are visualized as white gaps and can include insertions and deletions.
b LA1039 specific SNPs identified by SyRI comparison to Moneyberg-TMV, plotted in 10kb windows over the genomic position on the Moneyberg-TMV genome.

Supplementary Fig. 8

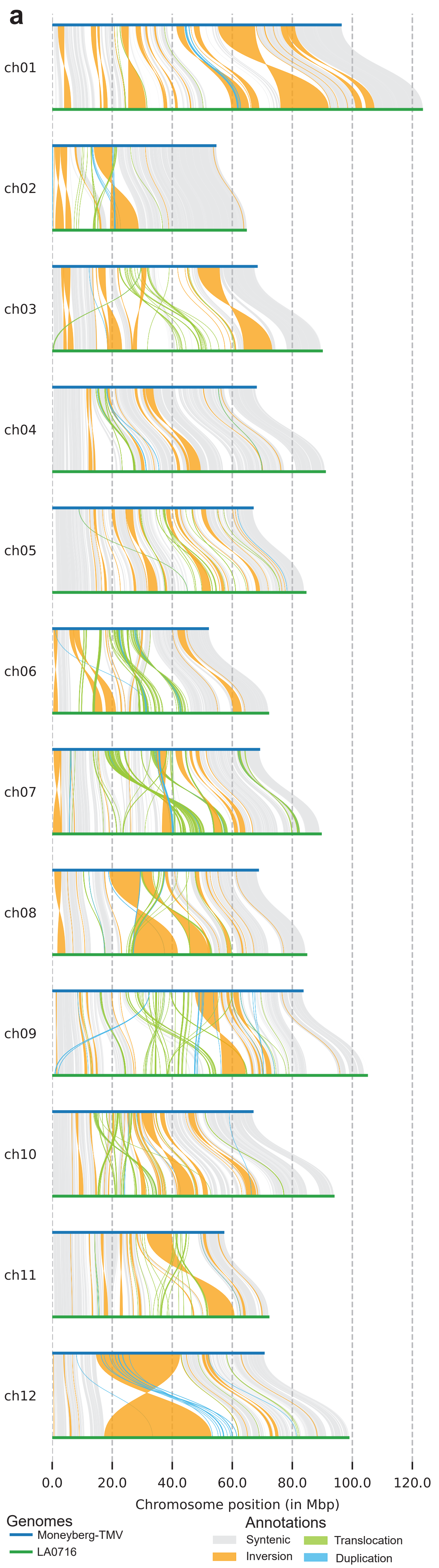

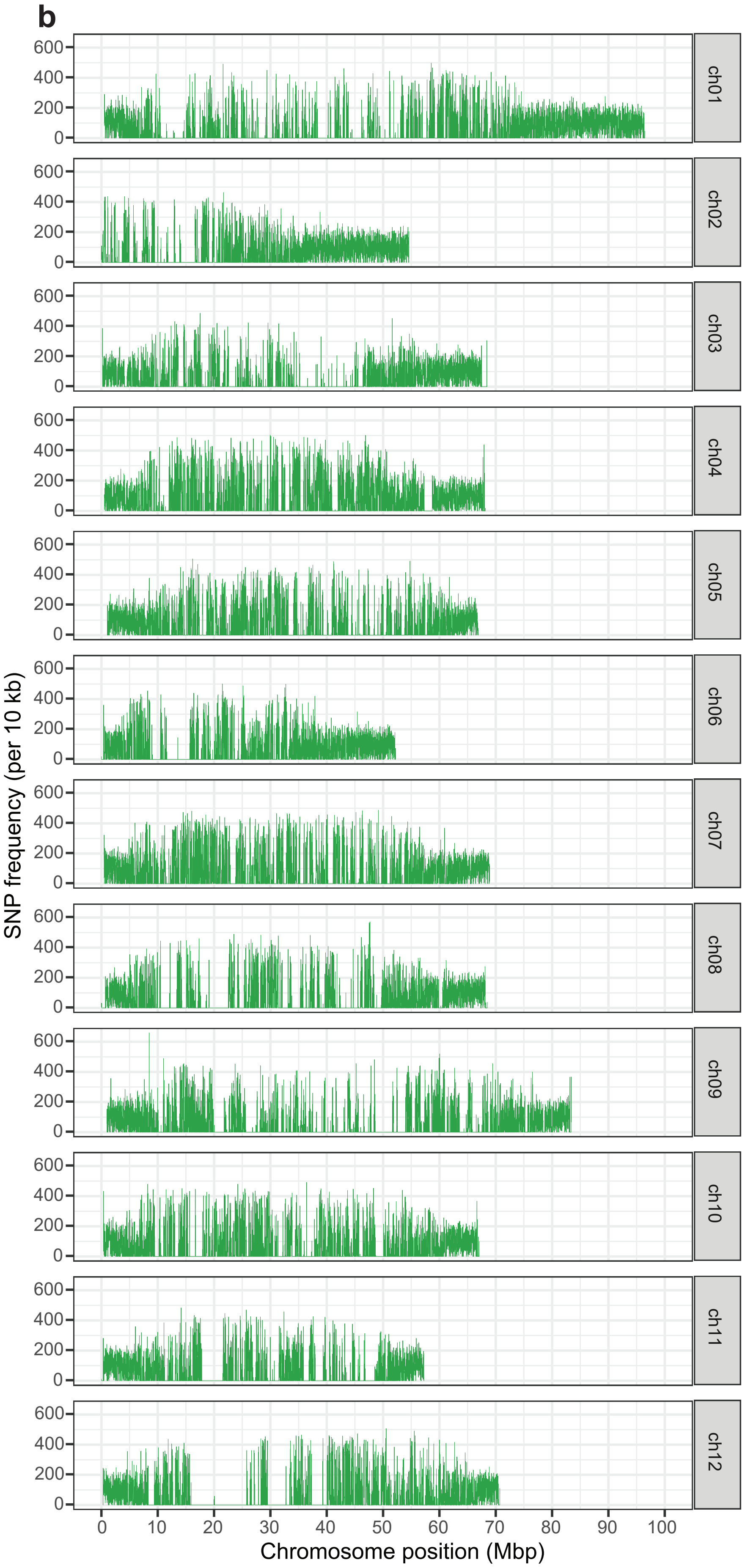

Genomic variation between Moneyberg-TMV (MbTMV) and LA0716 as identified by Synteny and Rearangement identifier (SyRI)
a Synteny and Rearangement plot (SyRI) between *S. lycopersicum* cv. Moneyberg-TMV and *S. pennellii* LA0716. Non-aligned or non-syntenic regions are visualized as white gaps and can include insertions and deletions.
b LA0716 specific SNPs identified by SyRI comparison to Moneyberg-TMV, plotted in 10kb windows over the genomic position on the Moneyberg-TMV genome.

Supplementary Fig. 9

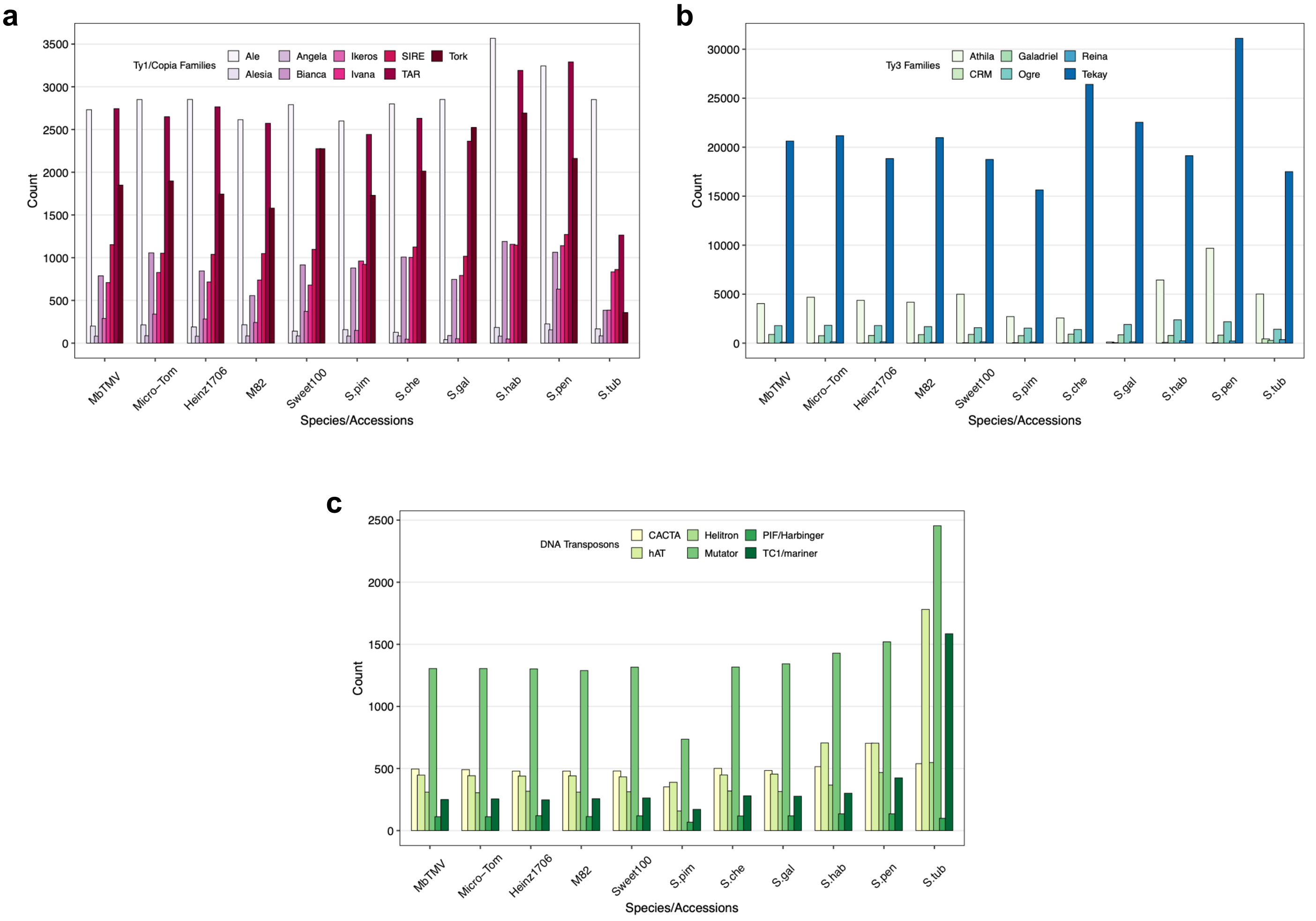

Abundance of high-quality LTR retrotransposon fragments and intact DNA transposons of the tomato pangenome. **a** and **b** Quantification of high-quality LTR retrotransposon fragments of the main *Ty1/Copia* and *Ty3* lineages. **c** Abundance of the intact DNA transposon elements of each lineage. The S. pim accession is LA1589.

Supplementary Fig. 10

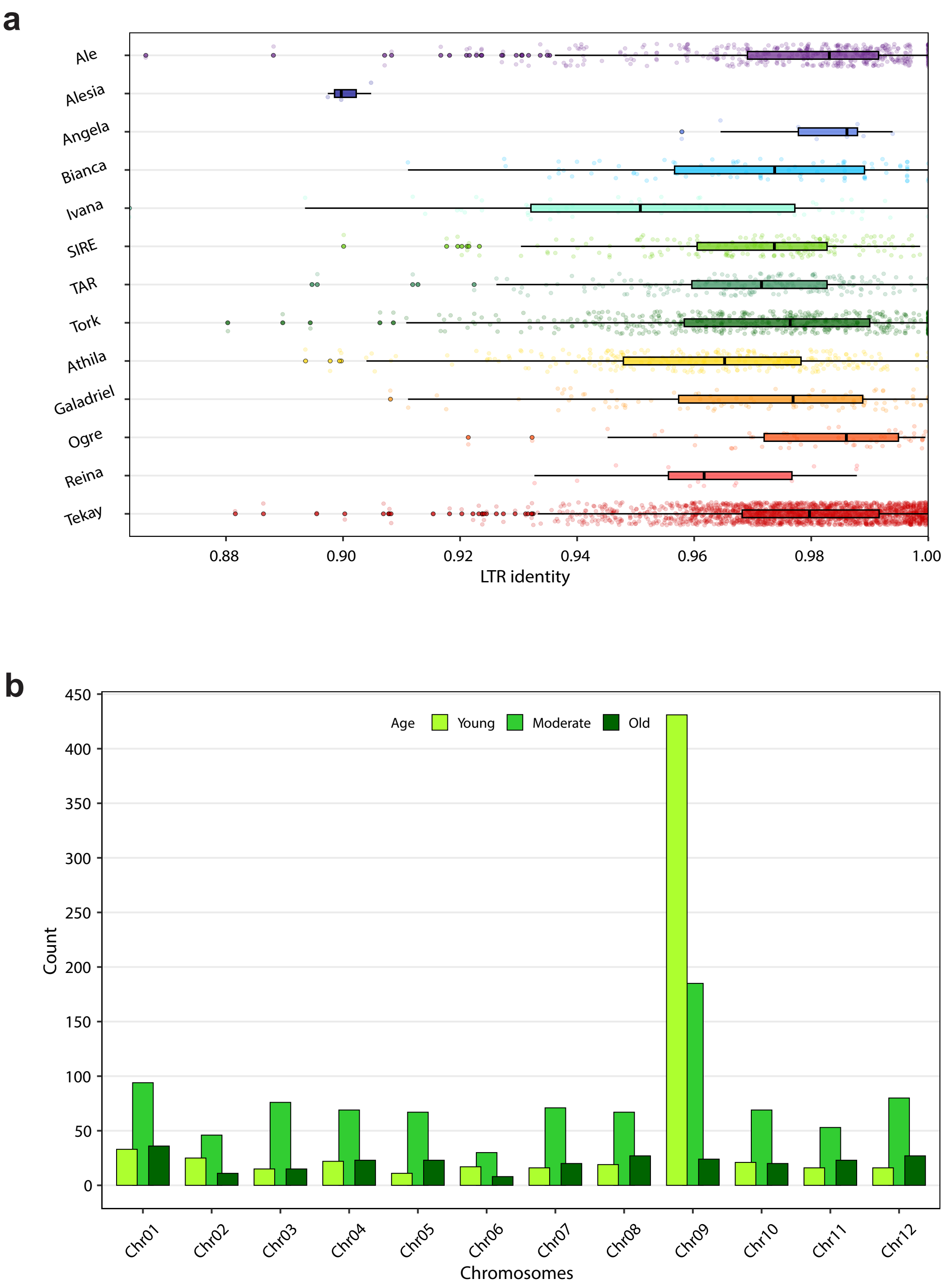

LTR identity of LTR retrotransposon lineages in MbTMV and position of Tekay elements on MbTMV chromosomes. **a** LTR identity of the main LTR retrotransposon lineages (Ty1/Copia and Ty3) in MbTMV. **b** Age-related abundance of Tekay elements on MbTMV chromosomes.

Supplementary Fig. 11

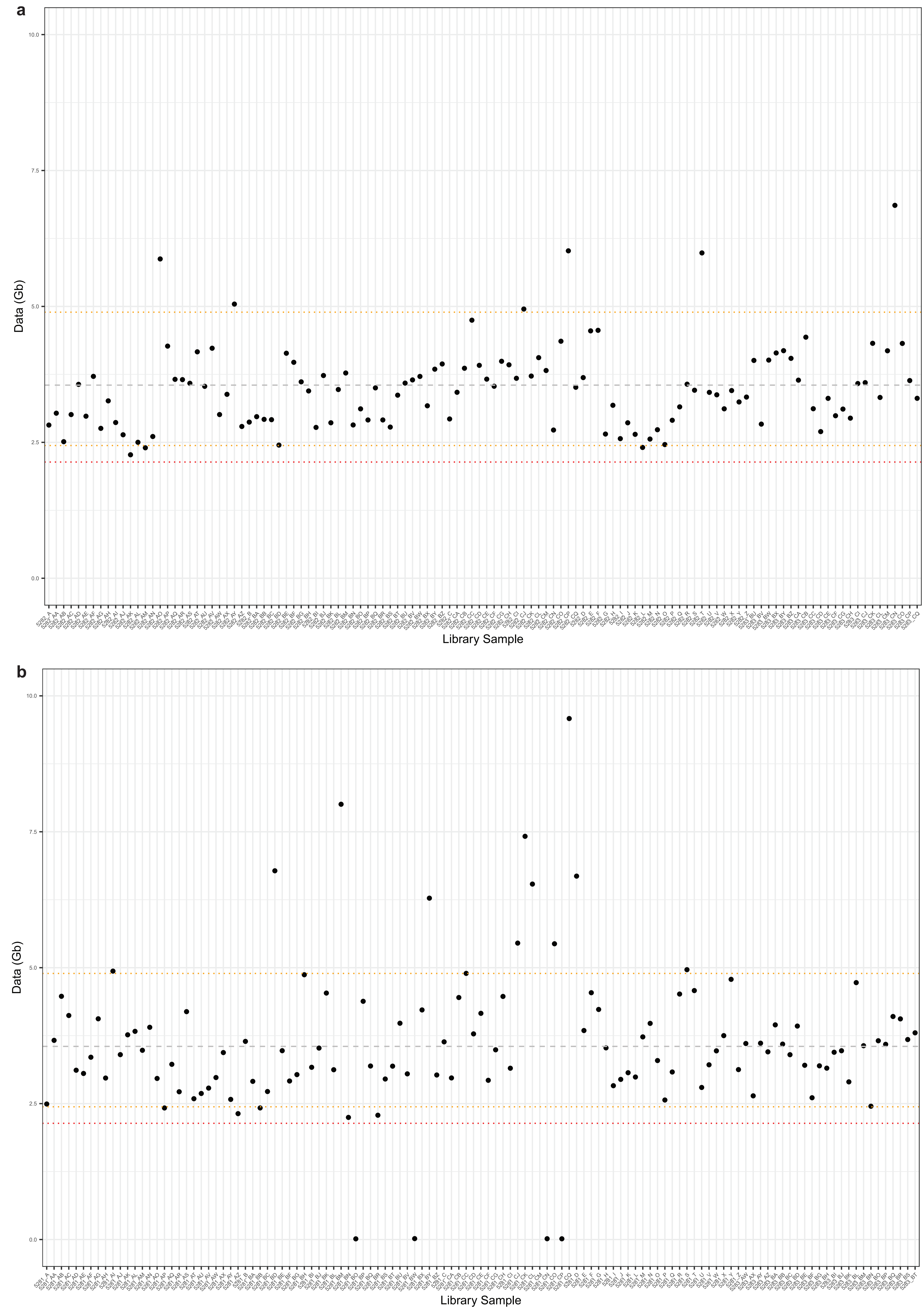

Generated sequencing data per Moneyberg-TMV x Micro-Tom F1 (MbTMV-MT) derived backcrossed individuals. **a** Female backcross samples, showing total data (Gb) generated per individual. **b** Male backcross samples, showing total data (Gb) generated per individual. Grey dashed line represent mean data. Yellow dashed lines represent 5% and 95% quantiles. Red dashed lines represent 2.5% and 97.5% percentiles. 4 Male backcross samples failed during sequencing.

Supplementary Fig. 12

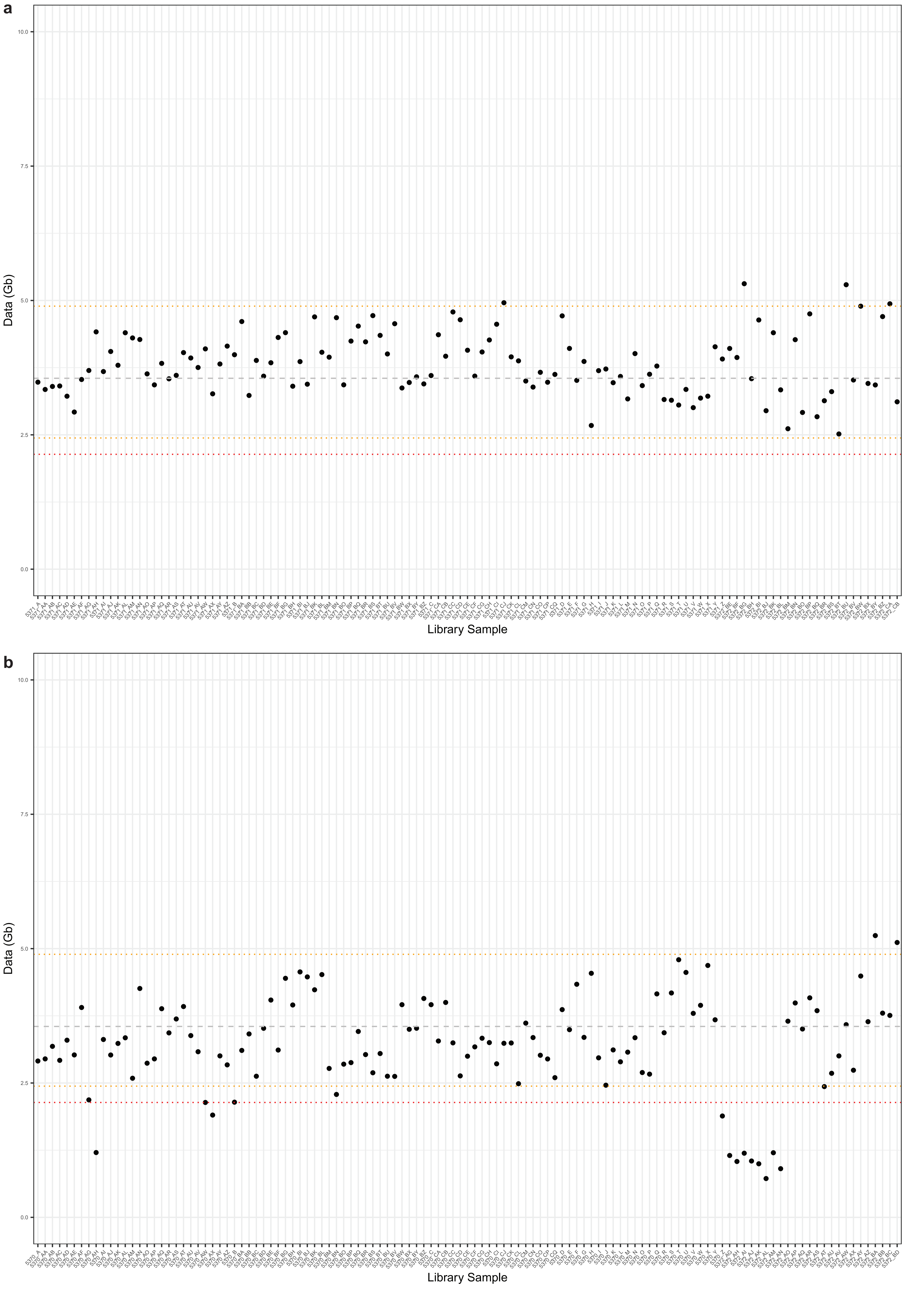

Generated sequencing data per Moneyberg-TMV x LA1039 F1 (MbTMV-S.che) derived backcrossed individuals. **a** Female backcross samples, showing total data (Gb) generated per individual. **b** Male backcross samples, showing total data (Gb) generated per individual.Grey dashed line represent mean data. Yellow dashed lines represent 5% and 95% quantiles. Red dashed lines represent 2.5% and 97.5% percentiles.

Supplementary Fig. 13

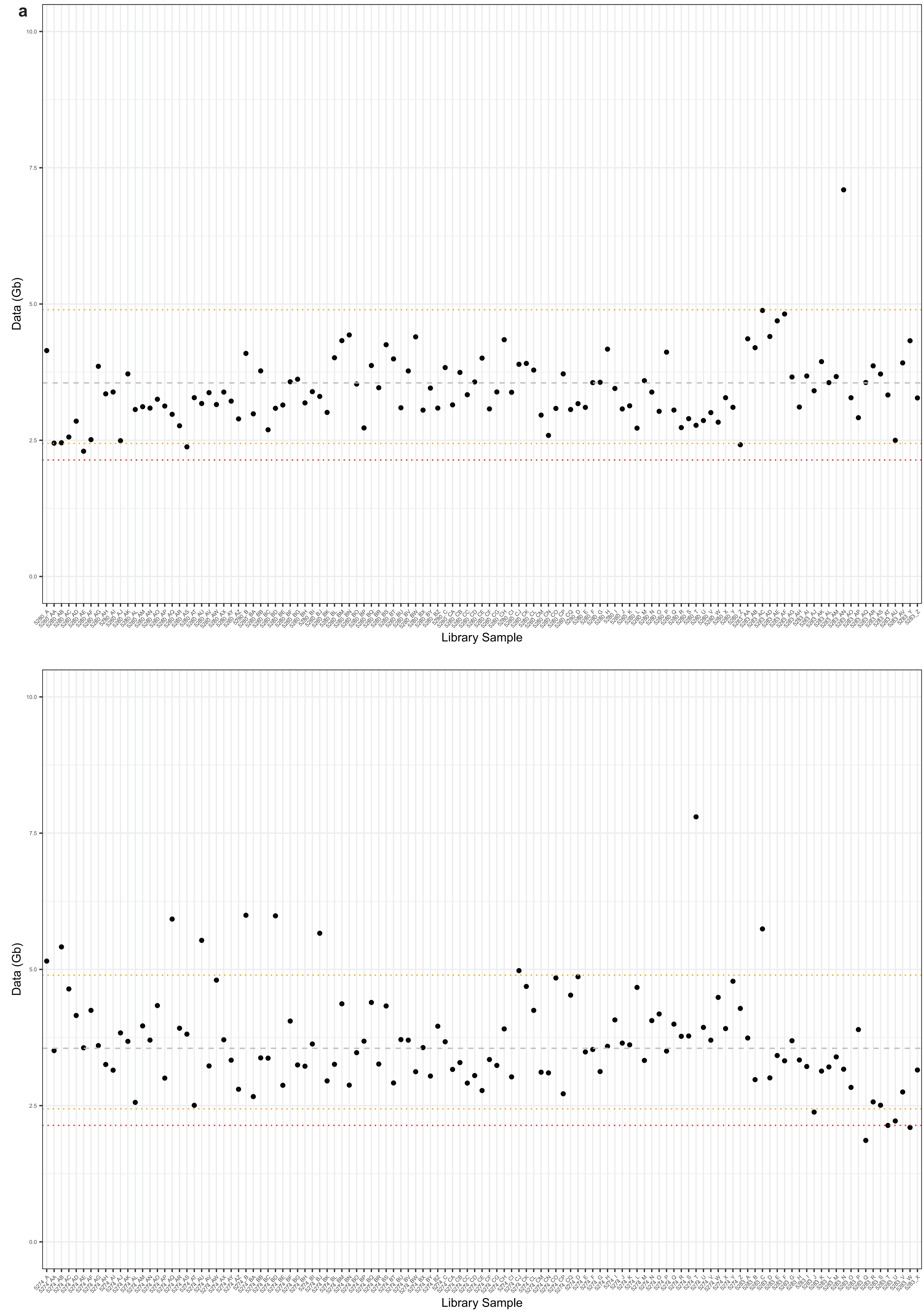

Generated sequencing data per Moneyberg-TMV x LA0716 F1 (MbTMV-S.pen) derived backcrossed individuals. **a** Female backcross samples, showing total data (Gb) generated per individual. **b** Male backcross samples, showing total data (Gb) generated per individual.Grey dashed line represent mean data. Yellow dashed lines represent 5% and 95% quantiles. Red dashed lines represent 2.5% and 97.5% percentiles.

Supplementary Fig. 14

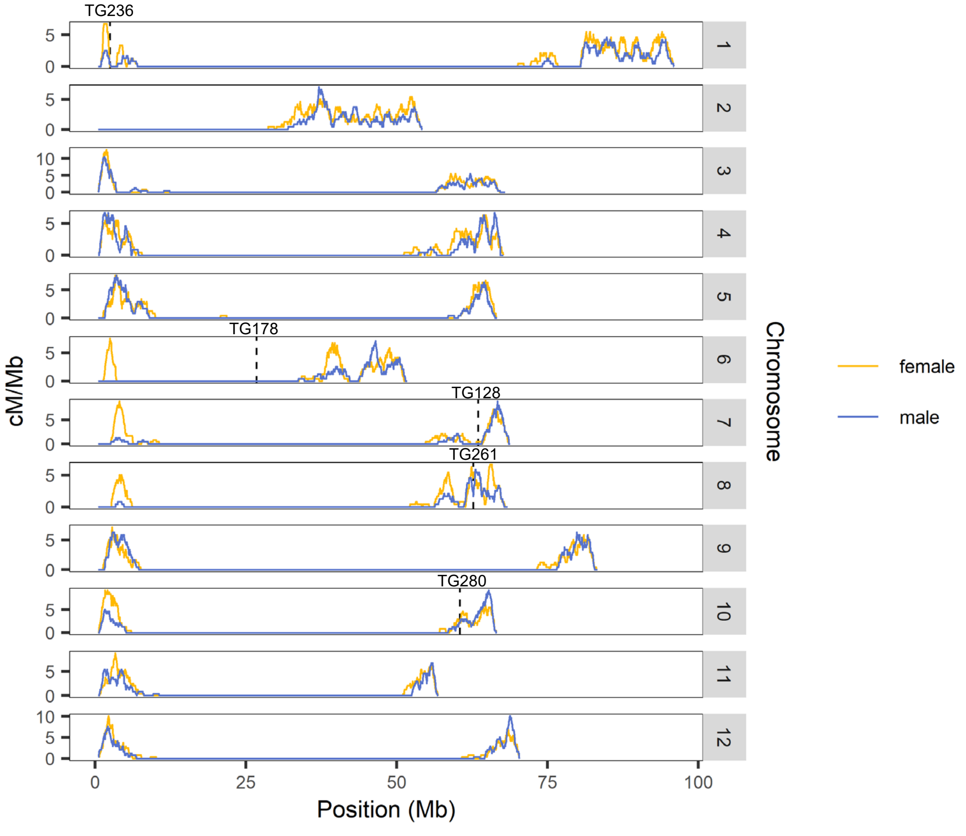

Female-specific recombination regions. Recombination landscape in male and female Mb-Pen backcross populations. The markers are based on the genetic maps constructed by de Vicente and Tanksley (1991). These markers are located upstream of the regions showing difference in genetic distance between male and female gametes, confirming the female-specific recombination regions in our backcross populations.

Supplementary Fig. 15

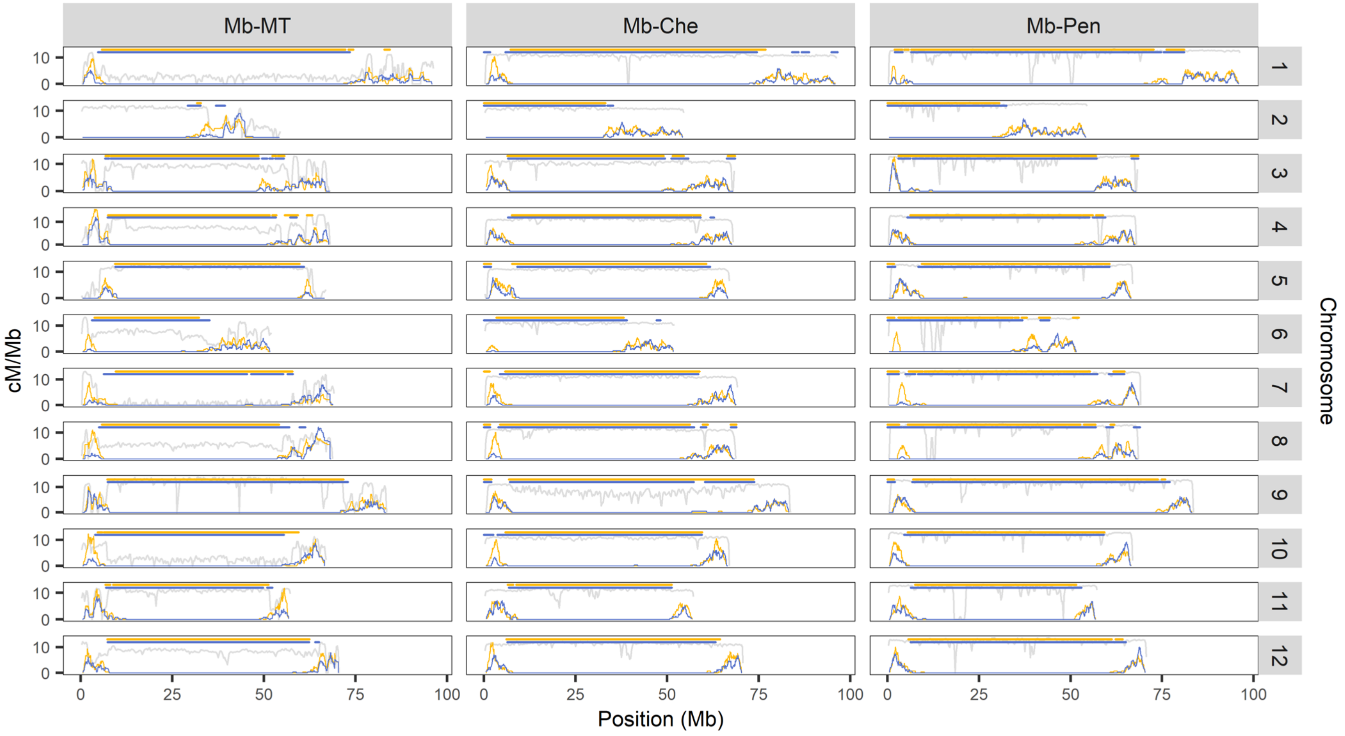

Recombination coldspots. Female (yellow) and male (blue) recombination landscape were plotted together with SNP distribution (gray). The top horizontal lines indicate the female and male recombination coldspots, which are mostly located in the pericentromeres.

Supplementary Fig. 16

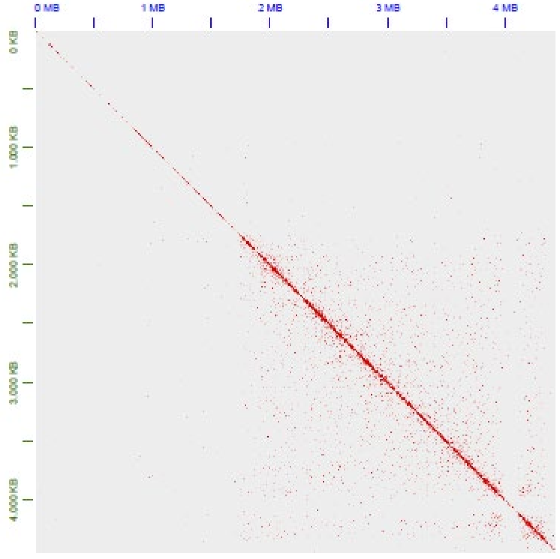

Inversion in chromosome 8. Hi-C map for an inversion causing crossover suppression between *MbTMV* and *S. pennellii*. The clean map confirms that the inversion is correct and not an assembly error or scaffolding artefact.

Supplementary Fig. 17

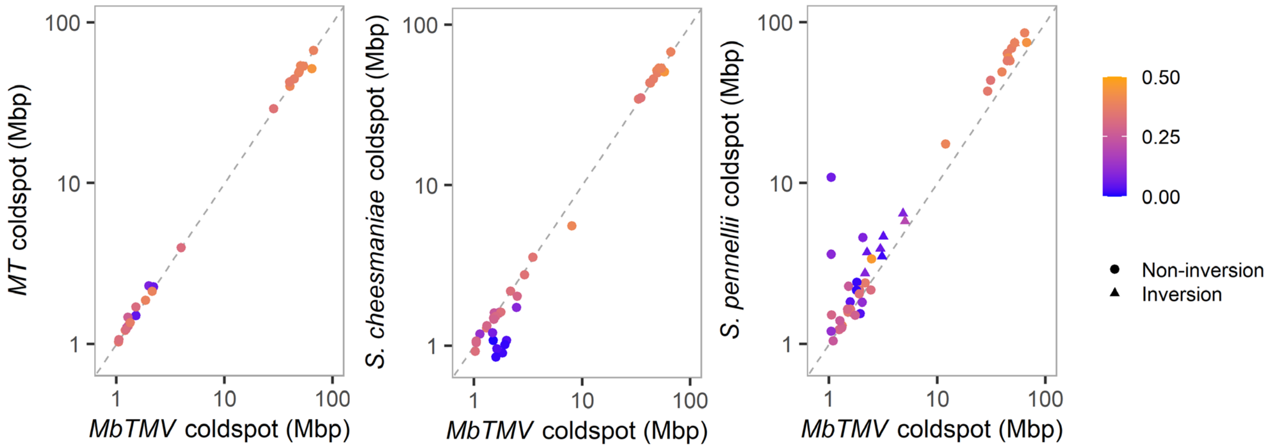

Coldspot comparison between parental genomes. Lengths of female coldspot regions relative to each parent. Deviation from the diagonal line indicates expansion or contraction of genomic segments, while the color indicates the total proportion of *Gyspy* and *Copia* elements relative to the MbTMV genome. We define inversion-associated coldspot as having more than 50% inversion coverage. Orange coldspots in the upper right of each plot are located in the pericentromeres.

Supplementary Fig. 18

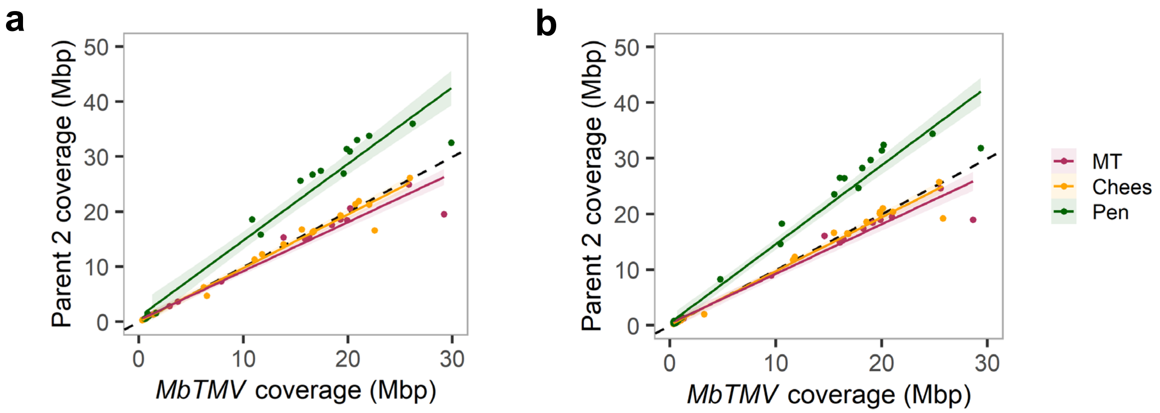

Retrotransposon copy changes in coldspots. Differential retrotransposon (i.e. Gypsy and Copia) content in **a** male and **b** female coldspot regions. Only coldspots with at least 25% retrotransposon coverage in MbTMV genome are included. Parent 2 is the parent crossed with MbTMV, differentiated by color. The broken diagonal line represents the equal parental copies of retrotransposons in the coldspot regions. Points above the diagonal indicate higher retrotransposon content in parent 2.

Supplementary Tables

Supplementary Table 1: Overview of the publicly available genomes used for pairwise genomic comparison and TE annotation in this study

Species	Accession	Genome size scaffolded on chromosomes (Mbp)	Accession number or Link	Citation
S. lycopersicum	Moneyberg-TMV	824.45	NCBI Bioproject: PRJEB44956	van Rengs et al., 2022
S. lycopersicum	Micro-Tom	812.44	https://datadryad.org/dataset/doi:10.5061/dryad.h9w0vt4qd	Wang et al., 2024
S. lycopersicum	Heinz1706	800.12	https://solgenomics.net/ftp/genomes/TGG/	Zhou et al., 2022
S. lycopersicum	M82	801.96	https://zenodo.org/records/6814693	Alonge et al., 2022
S. lycopersicum	Sweet-100	805.19	https://zenodo.org/records/6814693	Alonge et al., 2022
S. pimpinellifolium	LA1589	823.74	NCBI GenBank: GCA_034621305.1	Han et al., 2024
S. pimpinellifolium	LA2093	800.50	NCBI Bioproject: PRJNA607731	Wang et al., 2022
S. galapagense	LA0317	811.44	NGDC Genome Warehouse: SAMC605400	Yu et al., 2022
S. habrochaites	LA0407	893.57	NGDC Genome Warehouse: SAMC605399	Yu et al., 2022
S. tuberosum	DM8.1	737.87	http://www.bioinformaticslab.cn/pubs/dm8/	Yang et al., 2023

Supplementary Table 2: Statistics of PacBio HiFi sequencing data of *S. pennellii* LA0716 and *S. cheesmaniae* LA1039

Supplementary Table 2 shows the summary statistics of the PacBio HiFi sequencing datasets as calculated by Seqkit stats.

Accession	Library	Number of sequences	Total length	Minimum length	Mean length	Maximum length	Q1	Q2	Q3	sum_gap	N50	Q20(%)	Q30(%)
**LA0716**	5320.A	1,556,810	29,916,414,922	86	19,216.50	44,530	17,981	19,063	20,335	0	19,229	98.46	96.52
**LA0716**	5405.A	747,762	19,074,207,596	86	25,508.40	59,929	22,606	24,971	27,901	0	25,605	97.83	94.83
**LA1039**	5169.A	1,496,892	29,661,755,807	48	19,815.60	43,566	18,566	19,669	20,969	0	19,835	97.7	94.56
**LA1039**	5405.B	804,996	21,753,299,665	60	27,022.90	65,845	23,756	26,282	29,605	0	27,021	97.84	94.86

Supplementary Table 3: Statistics of *S. pennellii* LA0716 ONT sequencing data

Supplementary Table 3 shows the summary statistics of LA0716 ONT sequencing data as calculated by Seqkit stats.

Accession	Cell	Number of sequences	Total length	Minimum length	Mean length	Maximum length	Q1	Q2	Q3	sum_gap	N50	Q20(%)	Q30(%)
LA0716	Cell-1	6,248,270	27,654,234,087	2	4,425.90	319,192	540	1,318	3,479	0	17,686	82.24	70.77
LA0716	Cell-2	2,313,772	32,768,771,121	12	14,162.50	330,845	2,646	7,897	19,680	0	29,048	83.59	72.78
LA0716	Cell-3	2,005,259	22,595,261,204	1	11,268	282,104	1,571	5,749	15,039	0	24,967	86.5	76.75
LA0716	Cell-4	2,024,934	38,641,029,062	2	19,082.60	469,512	2,892	10,219	28,169	0	40,869	86.24	76.8
LA0716	Cell-5	2,619,041	43,127,741,818	4	16,467	386,073	1,745	7,298	23,893	0	39,607	86.49	77.16
LA0716	Cell-6	4,612,039	45,443,539,791	6	9,853.20	192,827	1,881	5,746	13,686	0	19,524	88.66	80.14
LA0716	Cell-7	2,171,619	36,943,275,247	11	17,011.90	368,751	1,825	8,470	25,580	0	37,745	88.37	79.74
LA0716	Cell-8	4,928,969	47,906,912,861	2	9,719.50	307,607	611	2,465	10,934	0	32,115	87.14	77.68
LA0716	Cell-9	5,733,819	37,511,690,014	1	6,542.20	289,982	400	1,218	5,312	0	28,469	89.46	81.33
LA0716	Cell-10	4,776,240	48,591,088,496	5	10,173.50	506,806	537	1,915	11,402	0	36,184	86.89	77.29

Supplementary Table 4: Statistics of Dovetail Omni-C sequencing data of *S. pennellii* LA0716 and *S. cheesmaniae* LA1039

Supplementary Table 4 shows the summary statistics of LA1039 and LA0716 Omni-C (Dovetail) data as calculated by Seqkit stats.

Accession	Library	Number of sequences	Total length	Minimum length	Mean length	Maximum length	Q1	Q2	Q3	sum_gap	N50	Q20(%)	Q30(%)
LA0716	5319.D	186,721,134	28,013,833,976	15	150.00	151	150	150	150	0	150	92.96	84.54
LA0716	5319.D	186,721,134	28,015,711,809	15	150	151	150	150	150	0	150	86.43	74.67
LA1039	5319.A	136,409,871	20,469,642,382	15	150.10	151	150	150	150	0	150	93.64	85.31
LA1039	5319.A	136,409,871	20,470,612,090	15	150.1	151	150	150	150	0	150	86.66	74.75

Supplementary Table 5: Statistics of Hifiasm assemblies of *S. cheesmaniae* LA1039 and *S. pennellii* LA0716

Supplementary Table 5 shows the summary statistics of LA1039 HiFI and LA0716 HiFi + ONT (>q90 >90kb) assemblies assembled using Hifiasm.

Accession	LA1039	LA0716
**Number of contigs**	836	423
**Number of contigs (>50kb)**	385	358
**Cumulative size (Mbp)**	862.66	1109.07
**N50 (Mbp)**	27.9	26.2
**N90 (Mbp)**	7.2	11
**L50**	11	14
**L90**	32	38
**Longest contig (Mbp)**	54	56.3

Supplementary Table 6: Comparison of *S. pennellii* LA0716 chromosome lengths

Supplementary Table 6 shows chromosome lengths (in Mbp) of each *S. pennellii* LA0716 chromosome assembled in this study and by Bolger *et al.* (2014).

Chromosome	LA0716, this study	LA0716 Bolger et al., 2014
ch01	123.5	109.3
ch02	64.9	59.8
ch03	90.2	75.4
ch04	91.2	77.2
ch05	84.7	78.0
ch06	72.3	60.7
ch07	89.8	79.3
ch08	85.0	70.5
ch09	105.2	84.1
ch10	94.1	82.5
ch11	72.4	66.2
ch12	99.1	83.3
Non-placed	36.7	63.1

Supplementary Table 7: Over-representation analysis of MapMan4 functional categories of genes that are in close proximity to Tekay elements

Genes in *S. pennellii* LA0716, *S. cheesmaniae* LA1039 and *S. lycopersicum* MbTMV genes were identified that intersected (+/- 2 Kbp) with intact, solo LTR, or fragments of Tekay elements on the same or opposite strand. Supplementary Table 7 shows the most over-represented Mapman4 functional categories for these genes. Over-representation analysis was performed using the one-sided Fisher's exact test.

MapMan4 category number	Context of Protein Function	#Genes of Interest IN MapMan4 category	#Genes of Interest NOT IN MapMan4 category	#Background Genes IN MapMan4 category	#Background Genes NOT IN MapMan4 category	Enrichment Factor	p-value	FDR-adjusted p-value
***S. pennellii* LA0716**								
10.4.1.1	Phytohormone action.cytokinin.biosynthesis.IP-type-cytokinin synthase *(IPT)	16	684	32	31966	22.86	1.96^{E-15}	1.05^{E-11}
11.3.2.7.4	Chromatin organisation.post-translational histone modification.histone acetylation.PEAT regulatory complexes.regulatory protein *(ARID2/3/4)	13	687	31	31967	19.17	5.01^{E-12}	1.07^{E-08}
14.5.3.5.2	RNA biosynthesis.DNA-binding transcriptional regulation.helix-turn-helix DNA-binding domain.ARID domain.transcription factor *(ARID3/4/6)	13	687	31	31967	19.17	5.01^{E-12}	1.07^{E-08}
11.3.2.7	Chromatin organisation.post-translational histone modification.histone acetylation.PEAT regulatory complexes	13	687	40	31958	14.86	6.81^{E-11}	1.04^{E-07}
***S. cheesmaniae* LA1039**								
10.4.1.1	Phytohormone action.cytokinin.biosynthesis.IP-type-cytokinin synthase *(IPT)	7	621	26	35493	15.23	1.33^{E-06}	6.14^{E-03}
14.5.6	RNA biosynthesis.DNA-binding transcriptional regulation.alpha-helix exposed by beta-structure.MADS/AGL transcription factor	11	617	161	35358	3.86	2.26^{E-04}	4.36^{E-01}
19.3.1	Protein physical control.endoplasmic reticulum protein translocation and insertion.Sec co-translocation	4	624	15	35504	15.08	2.84^{E-04}	4.36^{E-01}

	system.Signal Recognition Particle (SRP) complex							
***S. lycopersicum* cv. Moneyberg-TMV (MbTMV)**								
10.4.1.1	Phytohormone action.cytokinin.biosynthesis.IP-type-cytokinin synthase *(IPT)	5	660	19	34920	13.83	7.09^{E-05}	6.27^{E-01}
14.5.3.1.3	RNA biosynthesis.DNA-binding transcriptional regulation.helix-turn-helix DNA-binding domain.HOMEO domain.HD-ZIP IV-type transcription factor	6	659	40	34899	7.88	2.06^{E-04}	6.27^{E-01}
19.3.1.1	Protein physical control.endoplasmic reticulum protein translocation and insertion.Sec co-translocation system.Signal Recognition Particle (SRP) complex	4	661	13	34926	16.17	2.37^{E-04}	6.27^{E-01}

Supplementary Table 8: SNP-density (per kb) in *S. lycopersicum* cv. Micro-Tom, *S. cheesmaniae* LA1039 and *S. pennellii* LA0716 genomes, compared to *S. lycopersicum* cv. Moneyberg-TMV

Supplementary Table 8 shows the SNP density per kilobase derived from alignment of Micro-Tom, LA1039 and LA0716 PacBio HiFi data against the Moneyberg-TMV genome. SNP densities are shown per chromosome and whole genome.

	Micro-Tom	LA1039	LA0716
**Chromosome**	**HiFi**	**HiFi**	**HiFi**
**ch01**	0.28	4.21	22.83
**ch02**	4.10	5.00	22.39
**ch03**	1.74	3.82	22.44
**ch04**	1.79	5.73	23.67
**ch05**	8.37	4.76	25.34
**ch06**	0.96	5.08	21.79
**ch07**	0.52	5.11	24.53
**ch08**	0.22	6.13	23.20
**ch09**	15.93	16.96	23.84
**ch10**	0.18	4.40	24.15
**ch11**	4.26	6.93	21.07
**ch12**	1.12	6.79	24.49
**Genome**	3.29	6.24	23.31

Supplementary Table 9: Over-representation analysis of MapMan4 functional categories of genes in female recombination coldspots.

Over-representation analysis was performed using the one-sided Fisher's exact test.

**MapMan4 category number**	**Context of Protein Function**	**#Genes of Interest IN MapMan4 category**	**#Genes of Interest NOT IN MapMan4 category**	**#Background Genes IN MapMan4 category**	**#Background Genes NOT IN MapMan4 category**	**Enrichment Factor**	**p-value**	**FDR-adjusted p-value**
**MbTMVxPen (*S. pennellii* gene annotation)**								
14.5.6.1	RNA biosynthesis.DNA-binding transcriptional regulation.alpha-helix exposed by beta-structure.MADS/AGL transcription factor	124	11677	200	31798	1.68	5.44^{E-06}	1.45^{E-02}
10.4.1.1	Phytohormone action.cytokinin.biosynthesis.IP-type-cytokinin synthase *(IPT)	30	11771	32	31966	2.54	2.51^{E-04}	5.36^{E-01}
14.3.3.5.1	RNA biosynthesis.RNA polymerase II-dependent transcription.transcription co-activation.MEDIATOR complex.head module	32	11769	36	31962	2.41	3.02^{E-04}	5.36^{E-01}
**MbTMVxChees (*S. cheesmaniae* gene annotation)**								
14.5.6	RNA biosynthesis.DNA-binding transcriptional regulation.alpha-helix exposed by beta-structure.MADS/AGL transcription factor	83	10464	161	35358	1.74	4.84^{E-05}	1.11^{E-01}
10.4.1	Phytohormone action.cytokinin.biosynthesis.IP-type-cytokinin synthase *(IPT)	22	10525	26	35493	2.85	3.83^{E-04}	5.88^{E-01}

Supplementary Table 10: Over-representation analysis of MapMan4 functional categories of genes in female-enhanced recombination regions (FRRs)

Over-representation analysis was performed using the one-sided Fisher's exact test.

MapMan4 category number	Context of Protein Function	#Genes of Interest IN MapMan4 category	#Genes of Interest NOT IN MapMan4 category	#Background Genes IN MapMan4 category	#Background Genes NOT IN MapMan4 category	Enrichment Factor	p-value	FDR-adjusted p-value
**MbTMVxPen**								
14.5.10.6	RNA biosynthesis.DNA-binding transcriptional regulation.undefined DNA-binding domain.PLATZ transcription factor	17	3679	28	34911	5.74	3.63^{E-07}	1.28^{E-03}
15.1.6.7	RNA processing.spliceosome-mediated pre-mRNA splicing.spliceosome-associated non-snRNP factor activities.RNA splicing factor *(NSR)	15	3681	19	34920	7.46	1.58^{E-07}	1.28^{E-03}
20.2.1.3	Protein homeostasis.proteolysis.cysteine peptidase activities.Legumain-type asparaginyl endopeptidase *(AEP)	14	3682	17	34922	7.78	2.88^{E-07}	1.28^{E-03}
27.2.12.1	Multi-process regulation.Programmed Cell Death (PCD) system.vacuole-mediated cell death.cysteine proteinase *(VPE)	13	3683	16	34923	7.68	8.54^{E-07}	2.26^{E-03}
27.11.1.4	Multi-process regulation.UPR (Unfolded Protein Response) signalling.IRE1-bZIP60 pathway.regulatory E3 ubiquitin ligase *(PIR)	9	3687	15	34924	5.67	2.28^{E-04}	2.42^{E-01}
20.1.4.2.4.3	Protein homeostasis.ubiquitin-proteasome system.membrane-associated protein degradation.ER-associated protein degradation (ERAD).delivery to proteasome.ubiquitin-proteasome shuttle factor *(DSK2)	7	3689	9	34930	7.35	3.82^{E-04}	3.57^{E-01}
8.2.2.1.1	Secondary metabolism.phenolics biosynthesis.flavonoid biosynthesis.polyketide/acetate pathway.enoyl-CoA hydratase *(ECH)	6	3690	6	34933	9.45	4.24^{E-04}	3.57^{E-01}

16.5.2.3	RNA homeostasis.mRNA silencing.transacting siRNA pathway.regulatory chromatin remodeling factor *(CLSY3/4)	7	3689	10	34929	6.62	5.96^{E-04}	4.22^{E-01}
27.14.3.1	Multi-process regulation.MAP kinase cascade signalling.MAP3K-MEKK protein kinase activities.protein kinase *(MAPKKK13/14)	6	3690	7	34932	8.1	7.23^{E-04}	4.51^{E-01}
27.14.3.2	Multi-process regulation.MAP kinase cascade signalling.MAP3K-MEKK protein kinase activities.protein kinase *(MAPKKK15/16/17/18)	6	3690	7	34932	8.1	7.23^{E-04}	4.51^{E-01}
10.8.1.2.3	Phytohormone action.salicylic acid.biosynthesis.phenylalanine ammonia lyase pathway.benzyl benzoate 2-hydroxylase *(OSD3)	6	3690	9	34930	6.3	1.78^{E-03}	8.60^{E-01}
**MbTMVxChees**								
14.5.9.2.3	RNA biosynthesis.DNA-binding transcriptional regulation.beta-barrel DNA-binding domain.REM family.subgroup-C transcription factor	9	2867	10	34929	10.93	3.84^{E-06}	2.04^{E-02}
10.7.3.1	Phytohormone action.jasmonic acid.conjugation and degradation.jasmonoyl-amino acid synthetase *(JAR1)	7	2869	10	34929	8.5	1.44^{E-04}	2.70^{E-01}
14.5.6.1	RNA biosynthesis.DNA-binding transcriptional regulation.alpha-helix exposed by beta-structure.MADS/AGL transcription factor	27	2849	149	34790	2.2	3.77^{E-04}	4.00^{E-01}
9.7.1.1	Redox homeostasis.reactive electrophilic lipid homeostasis.oxylipin generation.9-lipoxygenase *(LOX1/5)	6	2870	9	34930	8.1	5.28^{E-04}	5.09^{E-01}
5.6.2.2	Lipid metabolism.lipid trafficking.endoplasmic reticulum-plasma membrane lipid transfer.lipid trafficking protein *(PLAT)	5	2871	6	34933	10.12	7.93^{E-04}	6.01^{E-01}
8.1.4.2	Secondary metabolism.terpenoid biosynthesis.terpene biosynthesis.mono-/sesquiterpene-/diterpene synthase	11	2865	37	34902	3.61	7.83^{E-04}	6.01^{E-01}

MbTMVxMT								
14.5.9.2.3	RNA biosynthesis.DNA-binding transcriptional regulation.beta-barrel DNA-binding domain.REM family.subgroup-C transcription factor	9	3759	10	34929	8.35	2.89^{E-05}	7.67^{E-02}
14.5.10.6	RNA biosynthesis.DNA-binding transcriptional regulation.undefined DNA-binding domain.PLATZ transcription factor	13	3755	28	34911	4.31	8.84^{E-05}	1.56^{E-01}
10.7.3.1	Phytohormone action.jasmonic acid.conjugation and degradation.jasmonoyl-amino acid synthetase *(JAR1)	8	3760	10	34929	7.42	1.43^{E-04}	2.16^{E-01}
20.1.4.2.4.3	Protein homeostasis.ubiquitin-proteasome system.membrane-associated protein degradation.ER-associated protein degradation (ERAD).delivery to proteasome.ubiquitin-proteasome shuttle factor *(DSK2)	7	3761	9	34930	7.21	4.26^{E-04}	5.02^{E-01}

Supplementary Table 11: List of the public short-read RNA-seq data used in the current study for evidence-based gene predictions for *S. lycopersicum* cv. Moneyberg-TMV (MbTMV) and *S. pennellii* LA0716 genomes.

Sample ID	Species
SRR25660662	Solanum lycopersicum
SRR25660663	Solanum lycopersicum
SRR25660664	Solanum lycopersicum
SRR25660665	Solanum lycopersicum
SRR25660668	Solanum lycopersicum
SRR25660679	Solanum lycopersicum
SRR25660680	Solanum lycopersicum
SRR25660666	Solanum lycopersicum
SRR24864451	Solanum lycopersicum
SRR24864452	Solanum lycopersicum
SRR24864453	Solanum lycopersicum
SRR24864457	Solanum lycopersicum
SRR24864458	Solanum lycopersicum
SRR24864459	Solanum lycopersicum
SRR24864460	Solanum lycopersicum
SRR24864461	Solanum lycopersicum
SRR24864462	Solanum lycopersicum
SRR24864463	Solanum lycopersicum
SRR24864464	Solanum lycopersicum
SRR24864465	Solanum lycopersicum
SRR24864469	Solanum lycopersicum
SRR24864470	Solanum lycopersicum
SRR24864471	Solanum lycopersicum
SRR24864474	Solanum lycopersicum
SRR24864475	Solanum lycopersicum

ERR3311252	Solanum pennellii
ERR3311249	Solanum pennellii
ERR3311250	Solanum pennellii
ERR3311251	Solanum pennellii
ERR3311253	Solanum pennellii
ERR3311254	Solanum pennellii
SRR27903361	Solanum pennellii
SRR27903365	Solanum pennellii
SRR27903360	Solanum pennellii
SRR27903363	Solanum pennellii
SRR27903362	Solanum pennellii
SRR27903364	Solanum pennellii
ERR2576914	Solanum pennellii
ERR2576913	Solanum pennellii
ERR2576918	Solanum pennellii
SRR21505619	Solanum pennellii
SRR21505620	Solanum pennellii
SRR21505626	Solanum pennellii
SRR21505623	Solanum pennellii
SRR21505624	Solanum pennellii
SRR21505625	Solanum pennellii
SRR21505627	Solanum pennellii
SRR21505622	Solanum pennellii
SRR18392547	Solanum pennellii
SRR18392543	Solanum pennellii
SRR18392545	Solanum pennellii
SRR18392551	Solanum pennellii
SRR18392559	Solanum pennellii
SRR18392548	Solanum pennellii
SRR18392560	Solanum pennellii

SRR18392561	Solanum pennellii
SRR18392550	Solanum pennellii
SRR18392556	Solanum pennellii
SRR18392557	Solanum pennellii
SRR18392562	Solanum pennellii
SRR18392544	Solanum pennellii
SRR18392546	Solanum pennellii
SRR18392549	Solanum pennellii
SRR18392552	Solanum pennellii
SRR18392553	Solanum pennellii
SRR18392554	Solanum pennellii
SRR18392555	Solanum pennellii
SRR18392558	Solanum pennellii
ERR2576913	Solanum pennellii
ERR2576914	Solanum pennellii
ERR2576918	Solanum pennellii
ERR3311249	Solanum pennellii
ERR3311250	Solanum pennellii
ERR3311251	Solanum pennellii
ERR3311252	Solanum pennellii
ERR3311253	Solanum pennellii
ERR3311254	Solanum pennellii
SRR18392543	Solanum pennellii
SRR18392544	Solanum pennellii
SRR18392545	Solanum pennellii
SRR18392546	Solanum pennellii
SRR18392547	Solanum pennellii
SRR18392548	Solanum pennellii
SRR18392549	Solanum pennellii
SRR18392550	Solanum pennellii

SRR18392551	Solanum pennellii
SRR18392552	Solanum pennellii
SRR18392553	Solanum pennellii
SRR18392554	Solanum pennellii
SRR18392555	Solanum pennellii
SRR18392556	Solanum pennellii
SRR18392557	Solanum pennellii
SRR18392558	Solanum pennellii
SRR18392559	Solanum pennellii
SRR18392560	Solanum pennellii
SRR18392561	Solanum pennellii
SRR18392562	Solanum pennellii
SRR21505619	Solanum pennellii
SRR21505620	Solanum pennellii
SRR21505622	Solanum pennellii
SRR21505623	Solanum pennellii
SRR21505624	Solanum pennellii
SRR21505625	Solanum pennellii
SRR21505626	Solanum pennellii
SRR21505627	Solanum pennellii
SRR27903360	Solanum pennellii
SRR27903361	Solanum pennellii
SRR27903362	Solanum pennellii
SRR27903363	Solanum pennellii
SRR27903364	Solanum pennellii
SRR27903365	Solanum pennellii

Supplementary Table 12: Statistics of PacBio Isoseq reads used in the current study for evidence-based gene predictions for *S. lycopersicum* cv. Moneyberg-TMV (MbTMV) and *S. pennellii* LA0716 genomes.

Supplementary Table 12 shows the summary statistics of LA0716 and MbTMV PacBio Isoseq data as calculated by Seqkit stats.

Species	Library	Number of sequences	Total length	Minimum length	Mean length	Maximum length	Q1	Q2	Q3	sum_gap	N50	Q20(%)	Q30(%)
Moneyberg-TMV	roots-anthers-leaf	4,705,644	5,123,548,961	51	1,088.80	7,059	690	937	1,375	0	1,230	99.6	99.12
LA0716_ pool 1	fruit-root-leaf-stem	11,485,181	44,688,801,326	44	3,891	25,242	2,952	3,669	4,599	0	4,061	99.36	98.54
LA0716_ pool 2	flower/bud-leaf-root	3,688,941	9,402,439,437	77	2,548.80	13,039	1,931	2,433	3,018	0	2,719	99.19	98.1

Supplementary Table 13: The adjusted ATHILAfinder parameters to detect *Tekay* elements in *Solanaceae* species

Order	Type	Input (e.g for *S. pennellii*)
**1**	Genome assembly	20230330_LA0716_genome.fasta
**2**	5’ LTR-PBS seed	lycopersicon_tekay_PBSjunction.fasta
**3**	PPT-3’ LTR seed	lycopersicon_tekay_PPTjunction.fasta
**4**	Seed window search	11,000 bp
**5**	Minimum internal domain length	2,000 bp
**6**	Maximum internal domain length	11,000 bp
**7**	Extend flanking window around internal domain	2,000 bp
**8**	Length of oligomers for criss-cross analysis	20 bp
**9**	Mismatches of oligomers	5 bp
**10**	Minimum LTR length	1,000 bp
**11**	Maximum LTR length	3,000 bp
**12**	Species code	Spen
**13**	LTR retrotransposon lineage	Tekay
**14**	Coverage for BLASTn rescue step	0.90
**15**	Minimum distance to identify internal junctions for BLASTn elements	500 bp
**16**	Maximum distance to identify internal junctions for BLASTn elements	3,000 bp

**17**	Mismatches of 5’ LTR-internal domain seeds	5
**18**	Mismatches of internal-3’ LTR domain seeds	5
**19**	Mismatches of oligomers (BLASTn step)	10
**20**	Coverage for BLASTn soloLTR annotation	0.98
**21**	HMM database	orfis.Ty3.updated.hmmdb
**22**	hmmscan e-value	0.01
