# Supplementary material for: Wild tomato genome assemblies reveal structural variants and repeat content act as recombination barriers
Source: Nat Commun. 2026 Jun 28;17:5590. doi: 10.1038/s41467-026-74784-5 (PMC13310191; doi:10.1038/s41467-026-74784-5)
Supplement: Supplementary file 2 — Description of Additional Supplementary Information [file 41467_2026_74784_MOESM2_ESM.pdf]

Description of Additional Supplementary Files

Supplementary Data 1: List of one-to-one orthologues between Solanum lycopersicum Heinz 1706 (ITAG4.0) and Solanum cheesmaniae LA1039

Supplementary Data 2: List of one-to-one orthologues between Solanum lycopersicum Heinz 1706 (ITAG4.0) and Solanum pennellii LA0716

Supplementary Data 3: Comparison of gene sets of S. lycopersicum Heinz 1706 (ITAG4.0), S. lycopersicum MbTMV, S. cheesmaniae LA1039 and S. pennellii LA0716 based on MapMan4 functional categories using the online tool Mercator4 v8

Supplementary Data 4: Recombination coldspots identified in tomato intraspecific and interspecific hybrid backcross populations

Supplementary Data 5: Female-enhanced recombination regions identified in tomato intraspecific and interspecific hybrid backcross populations
