# Supplementary material for: Wild tomato genome assemblies reveal structural variants and repeat content act as recombination barriers
Source: Nat Commun. 2026 Jun 28;17:5590. doi: 10.1038/s41467-026-74784-5 (PMC13310191; doi:10.1038/s41467-026-74784-5)
Supplement: Supplementary file 8 — Reporting Summary [file 41467_2026_74784_MOESM8_ESM.pdf]

nature portfolio

Corresponding author(s): Björn Usadel and Charles Underwood

Last updated by author(s): Jun 12, 2026

Reporting Summary

Nature Portfolio wishes to improve the reproducibility of the work that we publish. This form provides structure for consistency and transparency in reporting. For further information on Nature Portfolio policies, see our Editorial Policies and the Editorial Policy Checklist.

Statistics

For all statistical analyses, confirm that the following items are present in the figure legend, table legend, main text, or Methods section.

n/a	Confirmed	
☐	☒	The exact sample size (*n*) for each experimental group/condition, given as a discrete number and unit of measurement
☐	☒	A statement on whether measurements were taken from distinct samples or whether the same sample was measured repeatedly
☐	☒	The statistical test(s) used AND whether they are one- or two-sided<br>*Only common tests should be described solely by name; describe more complex techniques in the Methods section.*
☐	☒	A description of all covariates tested
☐	☒	A description of any assumptions or corrections, such as tests of normality and adjustment for multiple comparisons
☐	☒	A full description of the statistical parameters including central tendency (e.g. means) or other basic estimates (e.g. regression coefficient) AND variation (e.g. standard deviation) or associated estimates of uncertainty (e.g. confidence intervals)
☐	☒	For null hypothesis testing, the test statistic (e.g. *F*, *t*, *r*) with confidence intervals, effect sizes, degrees of freedom and *P* value noted<br>*Give P values as exact values whenever suitable.*
☒	☐	For Bayesian analysis, information on the choice of priors and Markov chain Monte Carlo settings
☒	☐	For hierarchical and complex designs, identification of the appropriate level for tests and full reporting of outcomes
☒	☐	Estimates of effect sizes (e.g. Cohen's *d*, Pearson's *r*), indicating how they were calculated

Our web collection on statistics for biologists contains articles on many of the points above.

Software and code

Policy information about availability of computer code

Data collection	DNA sequencing was carried out on a Pacific Biosciences Sequel II device (S. cheesmaniae LA1039 and S. pennellii LA0716), an Oxford Nanopore Technologies PromethION device (S. pennellii LA0716) and Omni-C libraries ((S. cheesmaniae LA1039 and S. pennellii LA0716) were sequenced on Illumina NextSeq 2000 and NovaSeq 6000 devices. Transposase-based libraries of the six backcross populations were sequenced on Illumina NextSeq 2000 and NovaSeq 6000 devices.
Data analysis	The software used in this study and specific parameters applied are extensively described in the methods section. The software used was:<br><br>k-mer Analysis Toolkit (KAT) hist v2.4.1, R v.4.2.3, GenomeScope2 v2, guppy versions 6.0.1, 6.0.2 and 6.1.1, seqkit stats v2.0.0, Hifiasm v0.16.1-r37559, Hifiasm v0.17.6-r46059, Burrows-Wheeler Aligner v0.7, SAMtools v1.9, BEDtools v2.30, Salsa v2.2, Juicebox v.1.11.08, Minimap2 2.24-r1122, D-GENIES version 1.4.0112, RagTag v2.1.0, pbmm2 v1.7.0, ChloroForge v1.0, Quast v5.0.2, GAAS v1.1.0, BUSCO v5.2.1, LTR retriever v2.9.0, GenomeTools v1.6.2, LTR_FINDER_parallel v1.2, Merqury v1.3, Mosdepth v0.3.2, Perl v.5.24.1, Helixer v0.3.3, Hisat2 v2.2.1, minimap2 v2.28, SAMtools v1.20, StringTie v2.2.3, Portcullis v1.2.4, Mikado v2.3.3, MMseqs2 v16-747c6, PSAURON v1.0.0, PSAURON v1.0.0, AGAT v1.4.0, Gffread v0.12.7, compleasm v0.2.6, OMArk v0.3.0, OrthoFinder v3.1.3, Mercator4 v8, GATK HaplotypeCaller v4.2.5.0, HTSJDK v2.24.1, Picard v2.25.4, BCFtools v1.15, SyRI v1.6, plotsr v0.5.4, BCT (R package) v.1.2, TRASH v2, circlize (R package) v.0.4.16, EDTA v.2.0.1, liftoff v1.6.1, TEsorter v.1.4.6, ATHILAfinder v1.0, MAFFT v.7.526, FastTree v.2.1.11, BEDTools v.2.31.1, trimgalore v0.6.6, BWA-MEM v0.7.17, GATK HaplotyperCaller v4.1.9.0, SAMtools v1.9, Sambamba markdup v0.8.1, MeiCOfi v1.0, hometools mapbp v1.0 and BCFtools v1.18.

For manuscripts utilizing custom algorithms or software that are central to the research but not yet described in published literature, software must be made available to editors and reviewers. We strongly encourage code deposition in a community repository (e.g. GitHub). See the Nature Portfolio guidelines for submitting code & software for further information.

Data

Policy information about availability of data

All manuscripts must include a data availability statement. This statement should provide the following information, where applicable:

- Accession codes, unique identifiers, or web links for publicly available datasets
- A description of any restrictions on data availability
- For clinical datasets or third party data, please ensure that the statement adheres to our policy

Raw HiFi, Omni-C and ONT sequencing data has been uploaded to the European Nucleotide Archive (ENA) under project codes: PRJEB62444 (S. cheesmaniae LA1039) and PRJEB62445 (S. pennellii LA0716). The genome assemblies and gene annotations can be found at the ENA under accessions CDWKDZ020000000 (S. cheesmaniae LA1039) and CDWKEA020000000 (S. pennellii LA0716). Illumina short-read sequencing data is available at the ENA for S. lycopersicum Moneyberg-TMV x S. lycopersicum Micro-Tom backcross populations (PRJEB101732), S. lycopersicum Moneyberg-TMV x S. cheesmaniae backcross populations (PRJEB101733) and S. lycopersicum Moneyberg-TMV x S. pennellii backcross populations (PRJEB101734). The S. lycopersicum Moneyberg-TMV Isoseq and S. pennellii LA0716 Isoseq data has been uploaded to the ENA under project codes PRJEB104324. Genomic variants between S. lycopersicum cv Moneyberg-TMV and the eleven other genomes as identified by SyRI, along with EDTA-based TE annotations, are freely available format at https://git.nfdi4plants.org/usadellab/Pennellii_Cheesmaniae_genomes.

Research involving human participants, their data, or biological material

Policy information about studies with human participants or human data. See also policy information about sex, gender (identity/presentation), and sexual orientation and race, ethnicity and racism.

Reporting on sex and gender	N/A
Reporting on race, ethnicity, or other socially relevant groupings	N/A
Population characteristics	N/A
Recruitment	N/A
Ethics oversight	N/A

Note that full information on the approval of the study protocol must also be provided in the manuscript.

Field-specific reporting

Please select the one below that is the best fit for your research. If you are not sure, read the appropriate sections before making your selection.

☒ Life sciences ☐ Behavioural & social sciences ☐ Ecological, evolutionary & environmental sciences

For a reference copy of the document with all sections, see nature.com/documents/nr-reporting-summary-flat.pdf

Life sciences study design

All studies must disclose on these points even when the disclosure is negative.

Sample size	For whole genome sequencing of S. cheesmaniae LA1039 (PacBio HiFi and Omni-C) and S. pennellii LA0716 (PacBio HiFi, ONT and Omni-C) the sequencing was carried out on single plants that were maintained in the greenhouse as cuttings. For the recombinant populations we respectively sequenced and analyzed 118 plants (S. lycopersicum MbTMV x S. lycopersicum Micro-Tom Female), 115 plants (S. lycopersicum MbTMV x S. lycopersicum Micro-Tom Male), 119 plants (S. lycopersicum x S. cheesmaniae Female), 119 plants (S. lycopersicum x S. cheesmaniae Male), 119 plants (S. lycopersicum x S. pennellii Female) and 119 plants (S. lycopersicum x S. pennellii Male).
Data exclusions	No data were excluded.
Replication	All attempts at replication were successful.
Randomization	During plant growth, the location of the different hybrid plant genotypes in the greenhouse was random.
Blinding	The watering and plant nutrient application was performed without knowledge of plant genotype by greenhouse gardeners. In any non-blinded analysis several authors reviewed the data to ensure robust data analysis had been carried out.

Reporting for specific materials, systems and methods

We require information from authors about some types of materials, experimental systems and methods used in many studies. Here, indicate whether each material, system or method listed is relevant to your study. If you are not sure if a list item applies to your research, read the appropriate section before selecting a response.

Materials & experimental systems

n/a	Involved in the study
[x]	[ ] Antibodies
[x]	[ ] Eukaryotic cell lines
[x]	[ ] Palaeontology and archaeology
[x]	[ ] Animals and other organisms
[x]	[ ] Clinical data
[x]	[ ] Dual use research of concern
[ ]	[x] Plants

Methods

n/a	Involved in the study
[x]	[ ] ChIP-seq
[x]	[ ] Flow cytometry
[x]	[ ] MRI-based neuroimaging

Dual use research of concern

Policy information about dual use research of concern

Hazards

Could the accidental, deliberate or reckless misuse of agents or technologies generated in the work, or the application of information presented in the manuscript, pose a threat to:

No	Yes
[x]	[ ] Public health
[x]	[ ] National security
[x]	[ ] Crops and/or livestock
[x]	[ ] Ecosystems
[x]	[ ] Any other significant area

Experiments of concern

Does the work involve any of these experiments of concern:

No	Yes
[x]	[ ] Demonstrate how to render a vaccine ineffective
[x]	[ ] Confer resistance to therapeutically useful antibiotics or antiviral agents
[x]	[ ] Enhance the virulence of a pathogen or render a nonpathogen virulent
[x]	[ ] Increase transmissibility of a pathogen
[x]	[ ] Alter the host range of a pathogen
[x]	[ ] Enable evasion of diagnostic/detection modalities
[x]	[ ] Enable the weaponization of a biological agent or toxin
[x]	[ ] Any other potentially harmful combination of experiments and agents

Plants

Seed stocks	S. cheesmaniae LA1039 and S. pennellii LA0716 seeds were obtained from TGRC (UC Davis, California, USA). Moneyberg-TMV seeds were kindly provided for research purposes by Cilia Lelivelt and Maarten Verlaan (Rijk Zwaan, Netherlands). Micro-Tom seeds were originally sourced from Tomato Growers Supply company (Ft. Myers, Florida, USA).
Novel plant genotypes	Moneyberg-TMV flowers were emasculated and pollinated with freshly extracted pollen of the other parent (e.g. Micro-Tom, S. cheesmaniae LA1039 and S. pennellii LA0716) 1 to 3 days after emasculation. To generate backcross populations, the three F1 hybrid genotypes were cultivated in the greenhouse and used for reciprocal crosses with S. lycopersicum cv. Moneyberg-TMV, apart from the S. lycopersicum Moneyberg-TMV x S. pennellii LA0716 female population where S. pennellii LA0716 was used as pollen donor.
Authentication	F1 hybrids were validated by phenotypic evaluation and eventually by genetic marker segregation in the backcross populations through whole genome sequencing.
