# Supplementary material for: Wild tomato genome assemblies reveal structural variants and repeat content act as recombination barriers
Source: Nat Commun. 2026 Jun 28;17:5590. doi: 10.1038/s41467-026-74784-5 (PMC13310191; doi:10.1038/s41467-026-74784-5)
Supplement: Supplementary file 9 — Transparent Peer Review file [file 41467_2026_74784_MOESM9_ESM.pdf]

nature portfolio

Peer Review File

Wild tomato genome assemblies reveal structural variants and repeat content act as recombination barriers

Corresponding Author: Professor Charles Underwood

This file contains all reviewer reports in order by version, followed by all author rebuttals in order by version.

Version 0:

Reviewer comments:

Reviewer #1

(Remarks to the Author)
Wild tomatoes are a valuable source of disease resistance and stress tolerance germplasm for tomato breeders. The authors of this manuscript describe the chromosome-level genome assemblies of two wild species, Solanum pennellii (accession LA0716) and Solanum cheesmaniae (accession LA1039). They investigated genomic variation between wild and cultivated tomato accessions, including megabase-scale structural variants and transposable element expansions. Particularly, using re-sequencing data from six large backcross populations derived from three hybrids with varying levels of DNA sequence divergence, the authors characterized crossover distribution and heterochiasmy in tomato. This resource is expected to enable genome-guided introgression of stress- and disease-resistance traits into cultivated tomato. Overall, the manuscript is well written and easy to follow. However, a few questions should be addressed.
1. The genome sequences and gene annotations provided in this study should be publicly accessible. The authors could share these data at NCBI (https://www.ncbi.nlm.nih.gov/) or NGDC (https://ngdc.cncb.ac.cn/). In addition, the authors are advised to provide, as supplementary files, the lists of homologous genes between these two wild species and the reference genome (accession Heinz 1706).
2. When investigating genomic structural variations, the authors should take into account the Solanum pimpinellifolium (e.g., LA1589 or LA2093, for which chromosome-scale genome sequences are available), the closest wild relative of the domesticated tomato. This could provide insights into structural changes during domestication.
3. The improved S. pennellii genome adds 146 Mb across the 12 chromosomes compared with the previous reference genome. Providing the genomic coordinates of these newly assembled regions would help researchers assess whether they impact the forward-mapped intervals.
4. The authors should provide the genomic coordinates of female-enhanced recombination and regions and coldspots in the supplementary materials. In addition, the relationships between these regions and previously reported trait loci are of particular interest. Given that LA0716 is frequently used for mapping important traits, such information would facilitate the precise utilization of key loci in future studies.
5. The journal name formatting in the references is inconsistent; for example, in references 55 and 56, the journal names are not abbreviated. The authors should carefully check and standardize all references.

Reviewer #2

(Remarks to the Author)
Overall evaluation
==================
This manuscript describes the high-quality genome production and analysis of two wild tomato species, Solanum pennellii and Solanum cheesmaniae as well some interesting studies about the sexual influence in the recombination rate. The manuscript is quite solid and the results are convincing and well supported from my point of view. One thing that I found missing is to link these results to the gene space and in a broader context to possible functions. In other words, the genome annotation is focused on TE and although it is described in detail how the gene models were annotated, no results were presented. In someways feels like something is missing although I understand that there is limitation on the manuscript extension and not everything that it was done can be presented. Overall, it is a good manuscript worthy to be read and for sure, with great interest for the tomato community of scientist and breeders.

Point-By-Point Manuscript Evaluation
======================================
A. Summary of the key results

The manuscript titled "Chromosome-scale Solanum pennellii and Solanum cheesmaniae genome assemblies reveal structural variants, repeat content and recombination barriers of the tomato clade" describes the generation and analysis of two high quality reference genomes for two tomato wild species (S. pennellii and S. cheesmaniae) as well as an experiment aiming to characterize the sexual biases during the chromosome recombination.
The results are summarized in the following points:
• Two high quality wild tomato genomes were produced. Both genomes are chromosome scale with high percentages of completeness (>99%). BUSCO scores were also high as well as the LAI. Compare with previously reported wild tomato genomes shows a quality improvement.
• A comprehensive SV catalog between these genomes and cultivated accessions were developed. As expected, S. pennellii showed a higher number of SV compared with the other wild tomato species, S. cheesmaniae. "Hirsutum" group tomato species showed a low synteny with cultivated tomatoes (S. pennelllii (46.3%) and S. habrochaites (36.2%)).
• Tomato species genomes have an explosion of LTR/Copia Tekay elements, especially high in S. pennellii. A detailed TE analysis revealed that the Ty3 Tekay element is especially abundant in the tomato genomes, being S. pennellii the highest of all. The TE divergency date indicates a recent proliferation of these elements.
• Female meiosis has a higher recombination rate than male. The analysis of a F1BC1 population showed a higher recombination rate for the female backcross populations.
• Recombination cold spots have a higher proportion of transposable elements. Recombination cold spots show a differential size when are compared across different species. This variation in size is associated with a variation in the TE content, especially for the Tekay element. Mb-S.pennellii cold spots have hemizygous genes.

Revised by Aureliano Bombarely on Jan. 30th, 2026

B. Originality and significance: if not novel, please include reference
----
The manuscript is original and novel.

C. Data & methodology: validity of approach, quality of data, quality of presentation.

The data and the methodology look fine, and it has been properly described.

D. Appropriate use of statistics and treatment of uncertainties
----
Yes, to my knowledge.

E. Conclusions: robustness, validity, reliability

The conclusions look valid and robust.

F. Suggested improvements: experiments, data for possible revision
----
The manuscript could benefit from the following improvements:
• Major – Poor reporting about the genome gene model annotation. Although the M&M describe the gene model annotation in detail including a solid QC (BUSCO, PSAURON, OMArk…), there is not a table summarizing these results.
• Major – Structural variants, TE analysis and recombination analysis is disconnected from the functional space. The manuscript does have an analysis of how SV, TE and recombination may impact the functional space, or in other words, how this is connected with genes. There are many questions that it could be explored such as: Is the Tekay explosion of S. pennellii afecting genes with some specific function associated with stress? Are the cold recombination spots enriched in genes with some specific functions?
• Major – No assemblies or annotations seems to be available. The data availability statement points the availability only of the raw data, but no assembly or annotation is available.
• Minor – Additional tables for supplementary data. It will be interesting to have a suppl. table (maybe an excel file) with some interesting information such as the coordinates for the cold spots as well.
• Discretionary – No TE event dating discussion. The Tekay element expansion is an intereting result of this manuscript. Unfortunatelly, it is somewhat disconnected from the evolutionary history of S. pennellii, for example hypothesizing if the proliferation of these elements is associated to the adaptation of this species to drought conditions or indeed happened after, being the adaptation to drough condition a ancestral property. Of course many of these points only can be clarify by other analysis and experiments, but it will be interesting to see these type of elements in the discussion.
• Discretionay – Assembly QC description could be improved. Even if the heterozygosity is low, it could be reported, maybe as suppl. information (e.g., GenomeScope Kmer figure). BUSCO values in the Table 1 are refering to the number of BUSCO hits rather than the %. Usually % are easy to interpret. At the same time, it will be useful to have the % of contigs/assembly that was scaffolded into chromosomes. No information about the if the mitochondrial and the plastid genomes were fully recovered.
• Discretionary – The manuscript could benefit from some additional round of editing. Some terms and formatting needs some corrections. Here some examples: L154 – "Confirmation" > "Conformation". Some species names are not in italics…

G. References: appropriate credit to previous work?

Yes, to my knowledge. There are some references that have not properly cited as Hosmani, P. S. et al. which does not mention the journal (BioRxiv). Other examples are Mahmoud, M. et al. and Nurk, S. et al.

H. Clarity and context: lucidity of abstract/summary, appropriateness of abstract, introduction and conclusions
----
The abstract looks adequate and summarize the manuscript well.

Version 1:

Reviewer comments:

Reviewer #1

(Remarks to the Author)
The authors have satisfactorily addressed my previous concerns and significantly improved the manuscript. I have no further comments on the revised version.

Reviewer #2

(Remarks to the Author)
The authors have resolved all the questions and concerns of my first revision, so I do not have any further concern. From my point of view, the manuscript is fine and ready to move to the next step.

Beyond the status of the manuscript revision, I would recommend to double check the annotations for S. lycopersicum (Moneyberg-TMV) and S. cheesmaniae (LA1039). They have 3000-3500 more gene models than S. pennellii with higher % of conserved missing genes and lower PSauron values. My experience is that this could be related with TE genes missidentified as genes. One option could be to look for IPR domains associated to TEs for the protein coding genes. In our group we use a tool called DETENGA that we developed to check this as part of GAQET pipeline if you are interested (https://www.protocols.io/view/structural-genome-annotation-qc-with-gaqet2-8epv5k5rnv1b/v1).

Reviewed by Aureliano Bombarely on 2026-05-07

Open Access This Peer Review File is licensed under a Creative Commons Attribution 4.0 International License, which permits use, sharing, adaptation, distribution and reproduction in any medium or format, as long as you give appropriate credit to the original author(s) and the source, provide a link to the Creative Commons license, and indicate if changes were made.
In cases where reviewers are anonymous, credit should be given to 'Anonymous Referee' and the source.
The images or other third party material in this Peer Review File are included in the article's Creative Commons license, unless indicated otherwise in a credit line to the material. If material is not included in the article's Creative Commons license and your intended use is not permitted by statutory regulation or exceeds the permitted use, you will need to obtain

permission directly from the copyright holder.
To view a copy of this license, visit https://creativecommons.org/licenses/by/4.0/

Reviewer #1 (Remarks to the Author):

Wild tomatoes are a valuable source of disease resistance and stress tolerance germplasm for tomato breeders. The authors of this manuscript describe the chromosome-level genome assemblies of two wild species, Solanum pennellii (accession LA0716) and Solanum cheesmaniae (accession LA1039). They investigated genomic variation between wild and cultivated tomato accessions, including megabase-scale structural variants and transposable element expansions. Particularly, using re-sequencing data from six large backcross populations derived from three hybrids with varying levels of DNA sequence divergence, the authors characterized crossover distribution and heterochiasmy in tomato. This resource is expected to enable genome-guided introgression of stress- and disease-resistance traits into cultivated tomato. Overall, the manuscript is well written and easy to follow. However, a few questions should be addressed.

We are pleased the referee appreciated our work on genomic variation in the tomato clade and the impact it has on meiotic recombination landscapes in breeding.

1. The genome sequences and gene annotations provided in this study should be publicly accessible. The authors could share these data at NCBI (https://www.ncbi.nlm.nih.gov/) or NGDC (https://ngdc.cncb.ac.cn/). In addition, the authors are advised to provide, as supplementary files, the lists of homologous genes between these two wild species and the reference genome (accession Heinz 1706).

We agree that sharing the data publicly is essential. The genome sequences were made public before the paper was submitted under project codes PRJEB62444 (*S. cheesmaniae* LA1039) and PRJEB62445 (*S. pennellii* LA0716), although we recognize that they were not so easy to find with the information we had provided in the "Data availability" section. We have now added this sentence in the data availability section:

"The genome assemblies and gene annotations can be found at the ENA under accessions CDWKDZ020000000 (*S. cheesmaniae* LA1039) and CDWKEA020000000 (*S. pennellii* LA0716)."

We have additionally uploaded datasets to Sol Genomics Network at Cornell University to be integrated by Prof Lucas Müller. Finally, most of the processed data sets are also available at https://git.nfdi4plants.org/usadellab/Pennellii_Cheesmaniae_genomes and https://doi.org/10.60534/pxhwb-83c17

To provide homologoues genes, we have used orthofinder to define orthologs between the reference *S. lycopersicum* ITAG 4.0, *S. cheesmaniae* LA1039 and *S. pennellii* LA0716 genomes, this is now in Supplementary Tables 7 and 8. In

addition, we performed comparisons among the gene sets in respect to the functional categories which is shown in Supplementary Table 9.

2. When investigating genomic structural variations, the authors should take into account the Solanum pimpinellifolium (e.g., LA1589 or LA2093, for which chromosome-scale genome sequences are available), the closest wild relative of the domesticated tomato. This could provide insights into structural changes during domestication.

Thank you for this suggestion. We had originally not included the mentioned *Solanum pimpinellifolium* genome assemblies as they were assembled using the legacy PacBio CLR data (pre-HiFi). On reflection we agree it is an oversight to not include those genomes as *Solanum pimpinellifolium* is the closest wild relative to the domesticated tomato. Both genomes have now been added to the structural variation analysis in Figure 2. These genomes further confirmed the inversion on the short arm of chromosome 9:

"A similar inversion on the short arm of chromosome 9 is also found in both *S. pimipinellifolium* accessions, *S. habrochaites* and *S. pennellii* (Fig. 2a)"

But we also identified private changes in *S. pimpinellifolium*:

"Within the pericentromeric regions, complex structural variation was found between the *S. lycopersicum* genomes and *S. pimpinellifolium*, with substantial variation also present between the two *S. pimpinellifolium* accessions themselves (Fig. 2a). Outside of the pericentromeres, when compared to the five *S. lycopersicum* genomes private inversions were found in *S. pimpinellifolium* LA1589 at the start of chromosome 2, on the short arm of chromosome 8 and on the long arm of chromosome 11 (Fig. 2a). Due to the singleton nature of these SVs future observation in independent assemblies would add credibility to their validity."

In addition, we have provided .vcf files of:

1) MbTMV assembly vs original *S. pimpinellifolium* LA1589
2) MbTMV assembly vs new *S. pimpinellifolium* LA2093

The SyRI .vcf files are available at https://git.nfdi4plants.org/usadellab/Pennellii_Cheesmaniae_genomes

Finally, TE annotation for *Solanum pimpinellifolium* (LA1589) has been added to Figure 3 using the same analytical techniques as the rest of the species in this study. The EDTA file is available at https://git.nfdi4plants.org/usadellab/Pennellii_Cheesmaniae_genomes

3. The improved S. pennellii genome adds 146 Mb across the 12 chromosomes compared with the previous reference genome. Providing the genomic

coordinates of these newly assembled regions would help researchers assess whether they impact the forward-mapped intervals.

Thank you for bringing this to our attention as we also want to make sure the information can be used by other researchers. As such we have provided the variant calls between the original *S. pennellii* LA0716 assembly and the new *S. pennellii* LA0716 assembly as .vcf files that were produced by SyRI.

In addition, we have provided .vcf files of:

1) MbTMV assembly vs original *S. pennellii* LA0716
2) MbTMV assembly vs new *S. pennellii* LA0716

The SyRI .vcf files are available at:
https://git.nfdi4plants.org/usadellab/Pennellii_Cheesmaniae_genomes

4. The authors should provide the genomic coordinates of female-enhanced recombination and regions and coldspots in the supplementary materials. In addition, the relationships between these regions and previously reported trait loci are of particular interest. Given that LA0716 is frequently used for mapping important traits, such information would facilitate the precise utilization of key loci in future studies.

We agree it is important to make the results re-usable by others. To this end we have generated:

1) A list of female-enhanced recombination regions in each hybrid (Supplementary Table 14)
2) A list of coldspot regions in each hybrid (Supplementary Table 12)
3) A table showing over-represented MapMan4 functional categories of genes in in the above regions (Supplementary Tables 13 and 15)

5. The journal name formatting in the references is inconsistent; for example, in references 55 and 56, the journal names are not abbreviated. The authors should carefully check and standardize all references.

Thank you for pointing this out, there were indeed several inconsistencies which we have now addressed throughout the manuscript.

Reviewer #2 (Remarks to the Author):

Overall evaluation
==================
This manuscript describes the high-quality genome production and analysis of two wild tomato species, Solanum pennellii and Solanum cheesmaniae as well some interesting studies about the sexual influence in the recombination rate. The manuscript is quite solid and the results are convincing and well supported from my point of view. One thing that I found missing is to link these results to the gene space and in a broader context to possible functions. In other words, the genome annotation is focused on TE and although it is described in detail how the gene models were annotated, no results were presented. In someways feels like something is missing although I understand that there is limitation on the manuscript extension and not everything that it was done can be presented. Overall, it is a good manuscript worthy to be read and for sure, with great interest for the tomato community of scientist and breeders.

Thank you, we are pleased you appreciated the combination of the genome analysis with recombination rate. The functional annotation of the gene space has now been more fully explained in the 7th paragraph of the results section "Chromosome-scale *Solanum pennellii* and *Solanum cheesmaniae* genome assemblies". We have also introduced text in the section "Transposable element annotation of the Lycopersicon clade reveals independent explosions of Tekay retrotransposons" that addresses how the TE content interacts with the gene content - see paragraphs 6 and 7.

Your specific points are more fully addressed below.

Point-By-Point Manuscript Evaluation
====================================
A. Summary of the key results

The manuscript titled "Chromosome-scale Solanum pennellii and Solanum cheesmaniae genome assemblies reveal structural variants, repeat content and recombination barriers of the tomato clade" describes the generation and analysis of two high quality reference genomes for two tomato wild species (S. pennellii and S. cheesmaniae) as well as an experiment aiming to characterize the sexual biases during the chromosome recombination.
The results are summarized in the following points:
• Two high quality wild tomato genomes were produced. Both genomes are chromosome scale with high percentages of completeness (>99%). BUSCO scores were also high as well as the LAI. Compare with previously reported wild tomato genomes shows a quality improvement.

Thank you!

• A comprehensive SV catalog between these genomes and cultivated accessions were developed. As expected, S. pennellii showed a higher number

of SV compared with the other wild tomato species, S. cheesmaniae. “Hirsutum” group tomato species showed a low synteny with cultivated tomatoes (S. pennellliii (46.3%) and S. habrochaites (36.2%)).
• Tomato species genomes have an explosion of LTR/Copia Tekay elements, especially high in S. pennellii. A detailed TE analysis revealed that the Ty3 Tekay element is especially abundant in the tomato genomes, being S. pennellii the highest of all. The TE divergency date indicates a recent proliferation of these elements.
• Female meiosis has a higher recombination rate than male. The analysis of a F1BC1 population showed a higher recombination rate for the female backcross populations.
• Recombination cold spots have a higher proportion of transposable elements. Recombination cold spots show a differential size when are compared across different species. This variation in size is associated with a variation in the TE content, especially for the Tekay element. Mb-S.pennellii cold spots have hemizygous genes.

Revised by Aureliano Bombarely on Jan. 30th, 2026

B. Originality and significance: if not novel, please include reference
----
The manuscript is original and novel.

C. Data & methodology: validity of approach, quality of data, quality of presentation.

The data and the methodology look fine, and it has been properly described.

D. Appropriate use of statistics and treatment of uncertainties
----
Yes, to my knowledge.

E. Conclusions: robustness, validity, reliability

The conclusions look valid and robust.

F. Suggested improvements: experiments, data for possible revision
----
The manuscript could benefit from the following improvements:
• Major – Poor reporting about the genome gene model annotation. Although the M&M describe the gene model annotation in detail including a solid QC (BUSCO, PSAURON, OMArk…), there is not a table summarizing these results.

Thank you, this was indeed an omission that we have corrected. The QC results using BUSCO, PSAURON and OMARK are now presented in Table 2. They are described in the manuscript in the 7th paragraph of the results section “Chromosome-scale *Solanum pennellii* and *Solanum cheesmaniae* genome assemblies” which reads:

“Furthermore, structural annotation of the genome assemblies was performed using *ab-initio* predictions for *S. cheesmaniae* whereas an integrated pipeline comprising both *ab-initio* and evidence-based approaches for MbTMV and for *S. pennellii* was used to control for repeat-driven mis-annotations. Annotation was followed by additional refinements (Supplementary Fig. 6), yielding consensus gene model sets for *S. cheesmaniae* (35,519), MbTMV (34,939), and *S. pennellii* (31,998) (Table 2). Benchmarking Universal Single-Copy Orthologs analysis indicated high completeness across all three annotated proteomes, at rates of 98.14% (*S. cheesmaniae*), 97.26% (MbTMV), and 98.38% (*S. pennellii*)[69]. Moreover, measures of the annotated proteome completeness and consistency regarding, respectively, the conserved homologs (HOGs) and known gene families of the ancestral lineage using OMArk confirmed the high quality of the predicted proteomes, identifying about 95.5–97.5% of expected HOGs and a large fraction of genes with consistent lineage placement (81.4% in *S. cheesmaniae*, 80.6% in MbTMV, and 96.3% in *S. pennellii*). Structural accuracy of the gene models, evaluated using PSAURON, further highlighted the robustness of the annotations, ranging from 77.3 in MbTMV to 97.8 in *S. pennellii*. Orthologous gene groups among the reference species *S. lycopersicum* (Heinz 1706 (ITAG4.0), *S. cheesmaniae* and *S. pennelli* were identified to extract orthologues (Supplementary Tables 7 and 8). Finally, we performed a functional annotation and cross-species comparisons among MbTMV, *S. cheesmaniae* and *S. pennelli* compared to the reference *Solanum lycopersicum* ITAG4.0 gene set, providing a systematic assessment of the gene presence–absence variation and the copy number differences among the species (Supplementary Table 9).”

- Major – Structural variants, TE analysis and recombination analysis is disconnected from the functional space. The manuscript does have an analysis of how SV, TE and recombination may impact the functional space, or in other words, how this is connected with genes. There are many questions that it could be explored such as: Is the Tekay explosion of S. pennellii affecting genes with some specific function associated with stress? Are the cold recombination spots enriched in genes with some specific functions?

Thank you, this was an excellent suggestion.

Given the *Tekay* expansion in tomato species and our previous experience designing TE-detecting algorithms, we decided to adapt ATHILAfinder (Primetis and Bousios, bioRxiv, 2026) to identify and characterise high-quality *Tekay* elements in the *Lycopersicon* clade. The signature sequences and parameters of ATHILAfinder were adjusted to reflect the characteristics of *Tekay* elements, which we identified and classified by EDTA and TEsorter, respectively. As a result, we detected more and higher-quality *Tekay* elements for downstream analysis, including their proximity to genes.

Using both EDTA and ATHILAfinder outputs, all the *Tekay* elements in MbTMV, *S. cheesemaniae*, and *S. pennellii* were examined to determine if they are found in proximity (+/- 2Kb) or within (introns) on the same or the opposite strand of the annotated genes in those species. We found the following which was introduced into the text:

“Due to the independent explosions of *Tekay* LTR retrotransposons in tomato wild relatives we investigated if these events are co-located with specific genes in these species. We thus

identified genes located within ±2 kb of, or intersecting with intact, solo LTR, or fragmented *Tekay* elements in MbTMV, *S. cheesmaniae* and *S. pennellii* and performed over-representation analysis of MapMan4 functional categories (Supplementary Table 10).This analysis revealed a pronounced enrichment of cytokinin biosynthesis *ISOPENTENYLTRANSFERASE* (*IPT*) genes[78] in *S. pennellii* (Fig. 3h). Similarly, in *S. cheesmaniae*, although with a lower magnitude compared to *S. pennellii*, *IPT* genes were enriched as well (Fig. 3h). In contrast, MbTMV only showed a non-significant tendency of enrichment of *IPT* genes (Fig. 3h). Further investigation revealed these *IPT* genes and *Tekay* elements are located in complex clusters on chromosomes 8 and 11 in the above species, and particularly the chromosome 11 cluster contributed to the proliferation of *IPT* genes co-located with *Tekay* elements in *S. pennellii* (Fig. 3i and Ext. Data Fig. 1). Consistent with the differential expansion of *Tekay* retrotransposons across species — being highest in *S. pennellii* —*Tekay* bursts might have contributed to the genomic enrichment and distribution of *IPT* genes in tomato genomes."

The results of this analysis, with focus on the enrichment of the *IPT* gene with *Tekay* elements, are depicted in Figures 3h and 3i.

Additionally, the top over-represented functional categories for the genes in proximity to *Tekay* elements across species are presented in Supplementary Table 10. We have also performed gene enrichment analysis in female recombination coldspots (Supplementary Table 13) and gene enrichment analysis in female recombination hotspots (Supplementary Table 15).

• Major – No assemblies or annotations seems to be available. The data availability statement points the availability only of the raw data, but no assembly or annotation is available.

As outlined in the reply to the first comment of referee one, the genome sequences were made public before the paper was submitted under project codes PRJEB62444 (*S. cheesmaniae* LA1039) and PRJEB62445 (*S. pennellii* LA0716), although we recognize that they were not so easy to find with the information we had provided in the "Data availability" section. We have now added this sentence in the data availability section:

"The genome assemblies and gene annotations can be found at the ENA under accessions CDWKDZ020000000 (S. cheesmaniae LA1039) and CDWKEA020000000 (S. pennellii LA0716)."

We have additionally uploaded datasets to Sol Genomics Network at Cornell University to be integrated by Prof Lucas Müller. Finally, most of the processed data sets are also available at: https://git.nfdi4plants.org/usadellab/Pennellii_Cheesmaniae_genomes and https://doi.org/10.60534/pxhwb-83c17

• Minor – Additional tables for supplementary data. It will be interesting to have a suppl. table (maybe an excel file) with some interesting information such as the coordinates for the cold spots as well.

As outlined in the reply to the fourth comment of referee one, we have generated:

1) A list of female-enhanced recombination regions in each hybrid (Supplementary Table 14)
2) A list of coldspot regions in each hybrid (Supplementary Table 12)
3) A table showing over-represented MapMan4 functional categories of genes in in the above regions (Supplementary Tables 13 and 15)

Now that these resources are available, we anticipate that they can be used by tomato breeding researchers to explore the regions in further details.

• Discretionary – No TE event dating discussion. The Tekay element expansion is an intereting result of this manuscript. Unfortunatelly, it is somewhat disconnected from the evolutionary history of S. pennellii, for example hypothesizing if the proliferation of these elements is associated to the adaptation of this species to drought conditions or indeed happened after, being the adaptation to drough condition a ancestral property. Of course many of these points only can be clarify by other analysis and experiments, but it will be interesting to see these type of elements in the discussion.

In our analysis regarding TE insertion near IPT genes we found that young *Tekay* insertions were found next to IPT genes in *S. pennellii*. In the discussion we have now included the following text:

"When analysing the co-location of *Tekay* elements with genes we found a strong over-representation of, and increase in copy number of, cytokinin biosynthesis *IPT* genes in the wild species, especially in *S. pennellii* (Fig. 3h, i). In particular young *Tekay* elements were identified close to *IPT* genes in *S. pennelli* but not in MbTMV and *Solanum cheesemaniae*. Cytokinin biosynthesis IPT proteins have been identified as rate-limiting factors in the production of cytokinin phytohormones and the modification of their expression can improve abiotic stress tolerance in diverse crop species, including cultivated tomato[78,99,100]. Given the variation in *IPT* gene copy number and TE presence/absence and insertion time in regulatory space, it will be interesting to explore the functional role of this amplified gene family in the abiotic stress tolerance of *S. pennellii* (accession LA0716)[3] and *Solanum cheesmaniae* (accession LA1039)[14]."

• Discretionay – Assembly QC description could be improved. Even if the heterozygosity is low, it could be reported, maybe as suppl. information (e.g., GenomeScope Kmer figure). BUSCO values in the Table 1 are refering to the number of BUSCO hits rather than the %. Usually % are easy to interpret. At the same time, it will be useful to have the % of contigs/assembly that was

scaffolded into chromosomes. No information about the if the mitochondrial and the plastid genomes were fully recovered.

Thank you GenomeScope2 k-mer spectra have been added to the Supplementary Figure 1 and the resulting low heterozygosity of about 0.2 % is referred to in the text:

"K-mer analysis of HiFi data revealed only a single clear peak for both accessions with residual heterozygosity estimated at ca. 0.2% indicating both accessions were highly homozygous (Supplementary Fig. 1)."

The BUSCO % is now listed in Table 1:

Accession	Moneyberg-TMV	Micro-Tom	LA1039	LA0716	LA2093	LA0716
Species	*S. lycopersicum*	*S. lycopersicum*	*S. cheesmaniae*	*S. pennellii*	*S. pimpinellifolium*	*S. pennellii*
Reference	van Rengs et al., 2022	Wang et al., 2024	This study	This study	Wang et al., 2020	Bolger et al., 2014
Number of sequences	12	12	12	12	12	12
Number of sequences (>50kb)	12	12	12	12	12	12
Cumulative size (Mbp)	824.45	812.44	814.47	1072.33	800.50	926.43
N50 (Mbp)	68.5	67.8	68.2	90.2	67.2	78
N90 (Mbp)	54.7	56.8	55.8	72.3	55.6	60.7
L50	6	6	6	6	6	6
L90	11	11	11	11	11	11
Longest sequence (Mbp)	96.5	96.3	96.7	123.5	95.5	109.3
Number of N's	0	4000	3300	4400	487215	71074709
Number of internal N-regions	0	40	33	44	373	41684
Raw LAI	9.67	8.42	8.64	12.43	8.49	3.24
LAI	15.3	14.05	14.27	18.06	14.12	8.87
Complete BUSCO (C)	5852 (98.35)	5851 (98.34)	5852 (98.35)	5857 (98.44)	5851 (98.34)	5827 (97.93)
Complete Single copy (S)	5747 (96.59)	5743 (96.52)	5741 (96.49)	5738 (96.44)	5744 (96.54)	5706 (95.9)
Complete Duplicated (D)	105 (1.76)	108 (1.82)	111 (1.87)	119 (2)	107 (1.8)	121 (2.03)
Fragmented (F)	12 (0.2)	12 (0.2)	12 (0.2)	14 (0.24)	12 (0.2)	15 (0.25)
Missing (M)	86 (1.45)	87 (1.46)	86 (1.45)	79 (1.33)	87 (1.46)	108 (1.82)
Total searched (Solanales)	5950	5950	5950	5950	5950	5950
QV	54.12	72.40	75.69	69.97	N.A.	N.A.
Completeness	99.10	99.22	99.17	99.17	N.A.	N.A.

We have recovered three chloroplast genomes (for *S. lycopersicum* MbTMV, *S. cheesmaniae* LA1089 and *S. pennellii* LA0716) and they have been made available at: https://git.nfdi4plants.org/usadellab/Pennellii_Cheesmaniae_genomes

• Discretionary – The manuscript could benefit from some additional round of editing. Some terms and formatting needs some corrections. Here some examples: L154 – "Confirmation" > "Conformation". Some species names are not in italics…

We have dealt with these issues and carefully checked the manuscript to remove any other mistakes.

G. References: appropriate credit to previous work?

Yes, to my knowledge. There are some references that have not properly cited as Hosmani, P. S. et al. which does not mention the journal (BioRxiv). Other examples are Mahmoud, M. et al. and Nurk, S. et al.

We have amended these citations and have carefully checked all of the other references and removed any remaining inaccuracies.

H. Clarity and context: lucidity of abstract/summary, appropriateness of abstract, introduction and conclusions

----

The abstract looks adequate and summarize the manuscript well.

Point by Point response

REVIEWERS' COMMENTS

Reviewer #1 (Remarks to the Author):

The authors have satisfactorily addressed my previous concerns and significantly improved the manuscript. I have no further comments on the revised version.

Thank you, we appreciated your comments.

Reviewer #2 (Remarks to the Author):

The authors have resolved all the questions and concerns of my first revision, so I do not have any further concern. From my point of view, the manuscript is fine and ready to move to the next step.

Thank you, we are grateful for your comments.

Beyond the status of the manuscript revision, I would recommend to double check the annotations for S. lycopersicum (Moneyberg-TMV) and S. cheesmaniae (LA1039). They have 3000-3500 more gene models than S. pennellii with higher % of conserved missing genes and lower PSauron values. My experience is that this could be related with TE genes missidentified as genes. One option could be to look for IPR domains associated to TEs for the protein coding genes. In our group we use a tool called DETENGA that we developed to check this as part of GAQET pipeline if you are interested (https://www.protocols.io/view/structural-genome-annotation-qc-with-gaqet2-8epv5k5rnv1b/v1).

Thank you for this comment. We have performed this analysis. We identified less than 1% of transcript models classified as potential TEs when looking for IPR domains associated to TEs using the DETENGA database with GAQET. This is now reported in the manuscript:

"Structural accuracy of the gene models, evaluated using PSAURON, further highlighted the robustness of the annotation (ranging from 77.3 in MbTMV to 97.8 in S. pennellii) and we identified less than 1% of transcript models were classified as potential TEs when looking for domains associated to TEs with GAQET"
